# The Introduction of a HuR-Binding Site in the 3′ UTR and the CD47 Cytoplasmic Tail Enhances SARS-CoV-2 S-Protein Expression in Cells

**DOI:** 10.3390/v18010137

**Published:** 2026-01-21

**Authors:** Ivan M. Pereverzev, Irina A. Bakhno, Kristina I. Yakovleva, Ilya S. Dovydenko, Evgeniya E. Burkova

**Affiliations:** Institute of Chemical Biology and Fundamental Medicine of the Siberian Branch of the Russian Academy of Sciences, 630090 Novosibirsk, Russia; pereverzevi1994@1bio.ru (I.M.P.); bakhno@1bio.ru (I.A.B.); yakovleva@1bio.ru (K.I.Y.); dovydenko_il@1bio.ru (I.S.D.)

**Keywords:** SARS-CoV-2 S-protein, cytoplasmic tail, 5′ and 3′ untranslated regions, HuR-binding site, CD47

## Abstract

In this study, we constructed plasmids to increase the overall expression level of the SARS-CoV-2 S-protein and its presentation on the cell surface. To this end, we designed a series of plasmid constructs encoding the SARS-CoV-2 S-protein with modifications to its cytoplasmic domain and containing various 5′ and 3′ untranslated regions. Our results confirmed the critical role of the S-protein cytoplasmic domain in limiting its localization to the cell surface. We confirmed that deletion of the 19 C-terminal amino acids, which contain an endoplasmic reticulum retrieval signal, significantly increased S-protein presentation on the cell surface. Furthermore, introducing the HuR-binding site from the CD47 3′ untranslated region and replacing the 19 C-terminal amino acids of the S-protein with the CD47 cytoplasmic tail significantly enhanced total S-protein expression compared to the wild-type S-protein and constructs with the 19-amino-acid deletion. Unfortunately, for the plasmid constructs bearing CD47 elements, their higher surface expression compared to the wild-type S-protein correlated with a high total protein expression level.

## 1. Introduction

Recent research has placed significant emphasis on the subcellular localization of the SARS-CoV-2 S-protein since understanding its cellular trafficking mechanisms is crucial for vaccine development. During synthesis a large amount of the S-protein accumulates in intracellular compartments [1,2].

Following vaccination with mRNA vaccines encoding the S-protein, only the surface-exposed antigen is recognized by B-cell receptors. This recognition triggers B-cell activation and subsequent antibody production. Consequently, efficient antigen translation is insufficient for the induction of high-efficacy immunity. A high level of antigen presentation on the cell surface is also necessary. Several constructs encoding the S-protein with various modifications to the cytoplasmic tail have currently been proposed [1,2,3]. Increasing S-protein presentation on the plasma membrane, virus-like particles, or extracellular vesicles may reduce the required vaccine dose. This, in turn, could reduce the potential side effects that occur after vaccination.

SARS-CoV-2 is an enveloped virus belonging to the *Coronaviridae* family. Its assembly occurs in the intermediate compartment between the endoplasmic reticulum (ER) and the Golgi apparatus, known as the ER–Golgi intermediate compartment (ERGIC) [4,5,6]. Coronavirus envelope proteins E, S, and M possess signal peptides [4,7].

S-protein plays a crucial role in combating coronavirus disease since it induces an immune response [8]. The receptor-binding domain of the S-protein binds to angiotensin-converting enzyme (ACE2) on the cell surface. The biosynthesis and maturation of SARS-CoV-2 S-protein involves several stages in different cell compartments. After synthesis on ribosomes and translocation into the ER lumen, the S-protein undergoes initial N-glycosylation [9]. Subsequent transport into the Golgi apparatus is accompanied by key modifications: N-linked oligosaccharide processing, O-glycosylation, and furin-mediated proteolytic cleavage at the S1/S2 site. S-palmitoylation of cysteines takes place in the ER or the Golgi apparatus depending on the type of DHHC palmitoyltransferase. After posttranslational modifications, the S-protein is transported to the ERGIC, where coronaviruses are assembled [6]. Mature viruses leave the cell via a secretory pathway. It should be noted that only a small part of the S-protein is transported to the plasma membrane of the host cell, where its presence induces the formation of a syncytium and the spread of infection [1,9].

**Figure 1:**
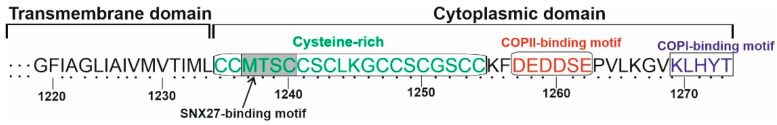
Schematic representation of the cytoplasmic tail of the S protein. Amino acids playing an important role in protein localization are highlighted.

Accumulation of all viral envelope proteins in the ERGIC is necessary for maturation of coronaviruses [10]. The transport of SARS-CoV-2 S-protein from the ER to the Golgi apparatus is carried out by COPII vesicles. Binding of COPII vesicles to the S-protein is mediated by a DEDDSE motif containing several acidic amino acids, which is located in the cytoplasmic tail (Figure 1). The S-protein contains a KLHYT motif at the C-terminus of its cytoplasmic domain. This motif is required for binding to COPI vesicles for retrograde transport from the Golgi apparatus to the ER [1]. However, the KLHYT motif has lower affinity for COPI proteins, leading to leakage of S-protein to the plasma membrane. Deletion of the last 19 amino acids of the S-protein significantly increases its presence at the cell surface and significantly enhance S-protein-mediated cell fusion [3,11]. The cytoplasmic tail of the SARS-CoV-2 S-protein also interacts with other host intracellular proteins, such as SNX27, which can transport it to the plasma membrane, and ERM family proteins, which are crosslinkers of the plasma membrane and actin cytoskeleton [1].

Introducing point substitutions into vaccine mRNAs that correspond to genetic variants of the virus provides an advantage in terms of a timely response to changes in the virus targeted by the vaccine. For effective antigen expression, mRNA requires several key components: a 5′ cap, a 5′ untranslated region (5′ UTR), a sequence encoding the antigen, a 3′ untranslated region (3′ UTR), and a poly(A) tail. 5′ and 3′ UTRs are important regulators of protein translation, and their selection is crucial for ensuring sufficient antigen production and effective vaccination. Previous studies on mRNA vaccine design have analyzed many endogenous UTRs for protein expression [12,13]. The 5′ and 3′ UTRs of alpha- and beta-globins are widely used to stabilize mRNA and enhance antigen translation efficiency [14]. The alpha-globin 5′ UTR provides higher translation efficiency than beta-globin 5′ UTR in cells [15]. Alternatively, 5′ UTRs can be designed using a de novo approach [13].

Various combinations of 5′ and 3′ UTRs are currently being studied in depth for use in mRNA vaccines and therapeutic mRNAs [16]. Studying the untranslated regions of mRNA could enable the widespread use of mRNA-based vaccines in response to the emergence of new pathogens such as SARS-CoV-2.

In this study, we developed plasmid constructs that enhance total S-protein expression and S-protein presentation on the cell surface. We designed a set of plasmid constructs encoding the SARS-CoV-2 S-protein with modifications to its cytoplasmic domain and containing various 5′ and 3′ untranslated regions. In addition, we incorporated a HuR-binding site from the CD47 3′ UTR and the CD47 cytoplasmic tail into the plasmid construct. We assumed that the introduction of a HuR-binding site (HuR-BS) and the cytoplasmic tail of CD47 would increase S-protein expression on the cell surface. It is known that the HuR-BS participates in the enhancement of CD47 protein exposure on the cell surface by recruiting specific transport factors [17,18]. Translocation of CD47 to the plasma membrane requires both its cytoplasmic tail and HuR-BS [17]. To evaluate the S-protein expression in cells, we performed Western blotting, immunofluorescence confocal microscopy, a cell-based ELISA, and a syncytia formation assay using the various constructs.

## 2. Materials and Methods

### 2.1. Cell Cultivation and Transfection

HEK293 cells were obtained from the Russian Cell Culture Collection (Institute of Cytology RAS, Saint Petersburg, Russia). HEK293FT cells stably expressing EGFP (enhanced green fluorescent protein) were kindly provided by Dr. Stepanov G.A. (SB RAS Institute of Chemical Biology and Fundamental Medicine, Novosibirsk, Russia). Vero cells were obtained from the cell culture collection of SRC VB “Vector” Rospotrebnadzor (State Research Center of Virology and Biotechnology “Vector” of Rospotrebnadzor, Koltsovo, Russia).

HEK293, HEK293FT-GFP, and Vero cells were cultured in a modified Minimum Essential Medium Eagle’s medium (Sigma, St. Louis, MO, USA) supplemented with 10% fetal bovine serum (Dia-M, Moscow, Russia) and penicillin/streptomycin at 37 °C and 5% CO_2_. Unless otherwise noted, cells were transfected using Lipofectamine 2000 (Invitrogen, Waltham, MA, USA). The ratio of Lipofectamine 2000 (µL) to DNA (µg) was 3:1. Lipofectamine 2000 (µL) was dissolved in DMEM (without fetal bovine serum and penicillin/streptomycin), incubated for 5 min at room temperature, and then DNA was added. The mixture was incubated for a further 25 min at room temperature before being added dropwise to cells that had been seeded the previous day.

### 2.2. Plasmid Design, Construction, and Synthesis

Details of the primers used in this study can be found in the Supplementary Materials.

**Figure 2:**
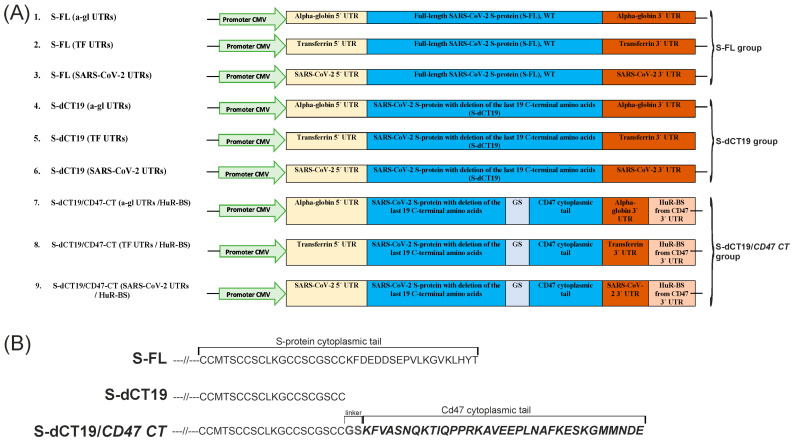
Schematic representation of plasmid constructs containing a 5′ UTR, the S-protein sequence, and a 3′ UTR. (**A**) Schematic representation of plasmid constructs containing a 5′ UTR, S-protein sequence (WT or with modifications of the cytoplasmic tail), and a 3′ UTR (with or without HuR-BS). The constructs contain the 5′ and 3′ UTRs of human alpha-globin, the 5′ and 3′ UTRs of human transferrin, or the 5′ and 3′ UTRs of SARS-CoV-2. The HuR-BS is a U-rich element from the CD47 3′ UTR. (**B**) Schematic representation of the cytoplasmic tail of the S-protein: S-FL—full-length S-protein (WT—wild-type, without mutations); S-dCT19—S-protein with a deletion of the last 19 C-terminal amino acids; S-dCT19/CD47-CT—S-protein with a deletion of the last 19 C-terminal amino acids and fused with the cytoplasmic tail of the transmembrane protein CD47 via a GS linker.

In this study, we used the pCMV14-3X-Flag-SARS-CoV-2 plasmid, which contains a codon-optimized S-protein sequence with a deletion of the last 19 C-terminal amino acids. The plasmid pCMV14-3X-Flag-SARS-CoV-2 S was a gift from Zhaohui Qian (Addgene plasmid # 145780; http://n2t.net/addgene:145780 (accessed on 5 January 2026); RRID: Addgene_145780). This plasmid encodes the S-protein of the Wuhan-Hu-1 SARS-CoV-2 strain. The 5′ and 3′ UTRs of human alpha-globin, human transferrin, and SARS-CoV-2, as well as *cis*-elements from the CD47 3′ UTR and a cytoplasmic domain with various modifications, were cloned into the pCMV14-3X-Flag-SARS-CoV-2 S plasmid. Figure 2 shows the maps of the pCMV14-3X-Flag-SARS-CoV-2 S plasmids with various 5′ and 3′ UTRs and different modifications to the S-protein cytoplasmic domain.

The nucleotide sequences of the 5′ and 3′ UTRs were obtained from the NCBI database. DNA fragments encoding the 3′ UTR of human alpha-globin, as well as the 5′ and 3′ UTRs of human transferrin and SARS-CoV-2, were assembled using polymerase cycling assembly. All oligonucleotides and fragments were synthesized in the Synthetic Biology Laboratory at the Institute of Chemical Biology and Fundamental Medicine, Siberian Branch of the Russian Academy of Sciences. The 5′ and 3′ UTRs sequences and primers are presented in Tables S1 and S2 in the Supplementary Materials.

In the first step, the 3′ UTR of transferrin was cloned into the pCMV14-3X-Flag-SARS-CoV-2 S plasmid at the BamHI site. Then, the 5′ UTR of alpha-globin and Sfr274I restriction site were included into the pCMV14-3X-Flag-SARS-CoV-2 S plasmid with the 3′ UTR of transferrin before the Kozak sequence using site-directed mutagenesis. The primers used are presented in Table S1 in the Supplementary Materials. Thus, the pCMV14-3X-Flag-SARS-CoV-2 S plasmid with the 5′ UTR of alpha-globin and the 3′ UTR of transferrin was obtained. The 5′ UTR of transferrin and SARS-CoV-2 were generated by annealing two DNA oligonucleotides that were inserted into the Sfr274I and HindIII sites. Then the 3′ UTRs of alpha-globin and SARS-CoV-2 were inserted at the XbaI and EcoRV sites. Thus, three plasmids were obtained: pCMV14-3X-Flag-SARS-CoV-2 S with the 5′ and 3′ UTRs of alpha-globin, transferrin, or SARS-CoV-2.

To obtain the full-length S-protein (wild-type), we introduced the 19 C-terminal amino acids into the above-mentioned plasmids using primers with a 5′ phosphate (see Table S2 in the Supplementary Materials). Pfu high-fidelity polymerase (Transgen, Beijing, China) was used to perform the mutagenesis. It should be noted that we used the S-protein without stabilization of the prefusion state in our study.

The next step was to generate plasmids containing a *cis*-element from the 3′ UTR of the human transmembrane protein CD47, as well as its cytoplasmic tail (KFVASNQKTIQPPRKAVEEPLNAFKESKGMMNDE). The sequence of the HuR-binding site (HuR-BS) from the CD47 3′ UTR is uuuaauagggugagcuugagag*uuuucuuucuuucuguuuuuuuuuuuuuuuu*gacuaauuucacaugcucuaa (the U-rich element is shown in italics). The HuR-BS was introduced at the end of the 3′ UTR sequence by PCR using the primers listed in Table S1 in the Supplementary Materials. The CD47 cytoplasmic tail was generated by annealing four DNA oligonucleotides. The sequence of the CD47 cytoplasmic tail and the 3′ UTR/HuR-BS were fused by overlap extension PCR (OE-PCR). Then, the resulting fragments were inserted into S-protein plasmids with a deletion of the last 19 C-amino acids at the BamHI and XbaI restriction sites.

The resulting plasmids were used to transform electrocompetent *E. coli* Top10 cells. Colonies were checked for the presence of the insert by PCR. DNA sequencing was performed at the SB RAS Genomics Core Facility. Plasmids were isolated using a Plasmid Midiprep 3.0 kit (Eurogen, Moscow, Russia) according to the manufacturer’s instructions.

### 2.3. Immunofluorescence Confocal Microscopy

The day prior to transfection, HEK293FT-EGFP cells (2 × 10^5^ cells/well) were plated in 24-well plates in cell culture medium. The cells were transiently transfected with 800 ng of plasmids and Lipofectamine 2000 (Invitrogen, Waltham (Massachusetts), USA). Transfection was performed overnight, and the medium was replaced with fresh medium the following morning. Twenty-four hours after transfection, the cells were transferred to chamber slides (SPL LifeSciences, Pocheon-si, Republic of Korea). After 36 h, the cells were fixed in 4% paraformaldehyde for 20 min at room temperature and washed three times with PBS. The cells were then permeabilized in PBST (0.1% Triton X-100 in PBS) for 5 min. Non-specific antibody binding was blocked by incubating the cells in 3% fetal bovine serum (FBS) (Dia-M, Moscow, Russia) for 1 h at room temperature and washed them three times with PBS. Next, the cells were incubated with primary polyclonal antibodies against S-protein (E-AB-V1006, Elabscience, Wuhan, China) overnight at 4 °C (1:1000 dilution), washed with PBS, and incubated with ElabFluor647-conjugated goat anti-rabbit antibody (1:300 dilution; E-AB-1075, Elabscience, Wuhan, China) for 30 min. The cells were washed with PBS, and then nuclei were stained with Hoechst 33342 (Neofroxx, Einhausen, Germany) for 15 min. The cells were washed with PBS and analyzed using a confocal microscope.

For *surface S-protein analysis*, the cells were not permeabilized. The cells were fixed with 4% paraformaldehyde, washed with PBS, and then blocked in 3% FBS in PBS. Antibody staining was performed as described for permeabilized cells; the antibody solutions were prepared in 3% FBS in PBS.

The slides were analyzed on an LSM 710 confocal microscope (Carl Zeiss Microscopy GmbH, Jena, Germany) using a 63× objective. ZEN blue edition 3.10 software package (Carl Zeiss, Jena, Germany) was used for image acquisition and processing.

### 2.4. Cell-Based ELISA

Vero cells (10^4^ cells/well, in triplicate) were plated in 96-well plates in cell culture medium the day prior to transfection. Cells were transiently transfected with 200 ng of plasmid and Lipofectamine 2000 (Invitrogen, Waltham, MA, USA) overnight, and the medium was replaced with fresh medium in the morning. At 48 h post-transfection, the cells were washed with PBS and fixed in 4% paraformaldehyde for 20 min at room temperature. Vero cells expressing the S-protein were stained with antibodies.

For *surface S-protein analysis*, the cells were not permeabilized. The cells were blocked with 3% FBS (Dia-M, Moscow, Russia) for 1 h at room temperature, followed by three washes with PBS. The antibodies were diluted in PBS containing 3% FBS. The cells were incubated with primary polyclonal antibodies against the S-protein (E-AB-V1006, Elabscience, Wuhan, China) overnight at 4 °C (1:1000 dilution), washed three times in PBS, and then incubated with HRP-conjugated goat anti-rabbit antibody (1:5000 dilution; E-AB-1194, Elabscience, Wuhan, China) for 30 min.

For *total S-protein analysis*, the cells were permeabilized in PBST (0.1% Triton X-100 in PBS) for 5 min after fixation. The cells were stained with antibodies using the protocol described above for non-permeabilized cells. The antibody solutions were prepared in PBST containing 3% FBS.

After incubation with secondary antibodies, the cells were washed with PBS, 100 μL of tetramethylbenzidine substrate (Euroimmun, Lübeck, Germany) were added to each well, and the cells were incubated for 5–10 min until the solution became a homogeneous blue color. The reaction was stopped by adding 100 μL of 0.1 M HCl. The absorbance was quantified at 450 nm with a reference wavelength of 620 nm using a Multiskan FC scanner (Thermo Fisher Scientific GmbH, Dreieich, Germany). The mean values of three replicates were normalized by subtracting the absorbance values of untransfected cells. Three independent biological experiments were performed.

### 2.5. Western Blot

HEK293FT-EGFP cells at approximately 70% confluence were transfected in 6-well plates. Two days after transfection, the cells were washed with PBS and detached from the plastic by incubating them with 2 mM EDTA in PBS for 15 min. The cell suspension was pelleted by centrifugation at 300× *g* for 5 min. The cells were lysed with RIPA buffer (1% NP-40, 150 mM NaCl, 0.1% SDS, and 50 mM Tris-HCl, pH 7.5) containing protease inhibitors (TransGen, Beijing, China) and phosphatase inhibitors (Servicebio, Wuhan, China). The cells were carefully pipetted and incubated for 30 min on ice, mixing occasionally. The resulting samples were centrifuged at 12,000× *g* for 30 min at 4 °C. The supernatant was stored at −20 °C. The total protein concentration in the lysates was determined using the BCA method (Abisence, Sirius, Russia) according to the manufacturer’s protocol. Optical density was measured using a Multiskan FC spectrophotometer (Thermo Fisher Scientific GmbH, Dreieich, Germany).

For electrophoretic analysis, the cell lysates were dissolved in loading buffer and incubated for 10 min at 95 °C. Twenty milligrams of total protein were loaded on the gel. The proteins were separated in a 10% PAGE gel using SDS-PAGE according to the Laemmli protocol. The Prestained Protein Ladder 10–250 kDa protein standard (G26619-250UL, Servicebio, Wuhan, China) was used as a marker. The separated proteins in the polyacrylamide gel were transferred to a PVDF membrane (WGPVDF45, Servicebio, Wuhan, China). The membrane was dried and blocked with 4% dry skim milk (Elabscience, Wuhan, China) in PBS-T (0.01% Triton X-100) for 30 min. S-protein staining was performed by incubating the membrane in primary antibodies (diluted 1:2000; E-AB-V1006, Elabscience, Wuhan, China) overnight at 4 °C. β-actin staining was performed by incubating the membrane in primary polyclonal rabbit antibodies (diluted 1:2000; E-AB-40517, Elabscience, Wuhan, China) for 1 h at room temperature. Then, the membrane was washed with PBS-T and incubated with HRP-conjugated goat anti-rabbit IgG (H + L) antibodies (diluted 1:5000; E-AB-1194, Elabscience, Wuhan, China) for 30 min. The membrane was washed three times with PBS-T. Detection was performed using diaminobenzidine (Neofroxx, Einhausen, Germany). Then, the PVDF membrane was washed several times in distilled water and dried. The membrane was scanned using a Molecular Imager Gel Doc XR+ (Bio-Rad, Hercules, CA, USA) and quantified using ImageQuant software 5.2 (GE Healthcare, Chicago, IL, USA). The Western blots were quantified using data from three independent experiments.

### 2.6. Cell–Cell Fusion Assay

Vero cells (acceptor) and HEK293FT-EGFP cells (donor) were seeded in a 12-well plate the day prior to transfection. HEK293FT-EGFP cells were transfected with 1 μg of plasmid and 3 μL of Lipofectamine 2000 (Invitrogen, Waltham, MA, USA) according to the manufacturer’s instructions. Twelve hours after transfection, the donor cells were detached and mixed with acceptor cells at a 1:1 ratio, and then plated in 35 mm microscopy dishes (Jet Biofil, Guangzhou, China). After 20 h of co-culture, nuclei were stained with Hoechst 33342 (Neofroxx, Einhausen, Germany) for 15 min. The cells were washed with PBS. The images were acquired with an LSM 710 confocal microscope (Carl Zeiss Microscopy GmbH, Jena, Germany) using a 20× objective in tile-scan mode. Nine fields were captured in tile-scan mode, which were merged during the imaging process with three horizontal and three vertical overlaps. ZEN blue edition 3.10 software (Carl Zeiss, Jena, Germany) was used to acquire and process the images. Syncytia were counted using ImageJ 1.54p.

### 2.7. MTT Assay

HEK293 and Vero cells (10^4^ cells per well, triplicates) were plated in 96-well plates in cell culture medium. After 24 h, transfection was performed with 200 ng of plasmids and Lipofectamine 2000 (Invitrogen, Waltham, MA, USA) according to the manufacturer’s protocol. Transfection was carried out overnight, and the medium was replaced with fresh medium in the morning. After 48 h, an MTT assay was performed. The culture medium was removed, 10 µL of MTT (5 mg/mL in PBS) was added to each well, and the cells were incubated for 2–4 h at 37 °C. The medium was removed and the dark purple formazan crystals were dissolved in 100 µL of isopropanol. The optical density of the solution was measured using a Multiskan FC scanner (Thermo Fisher Scientific GmbH, Dreieich, Germany) at two wavelengths: 570 nm and 620 nm (to subtract background absorption). The results are presented as percentages relative to the control value. Control cells were incubated with Lipofectamine 2000 only. Three independent biological experiments were performed.

### 2.8. Statistics and Reproducibility

Statistical analyses were performed using GraphPad Prism 8.0. All data are plotted as the mean and standard deviation (error bars) unless otherwise indicated. The data were analyzed using one-way ANOVA followed by Tukey’s HSD test. *p* < 0.05 was considered statistically significant.

## 3. Results

### 3.1. Generation of Plasmid Constructs Encoding Different S-Protein Variants and Containing 5′ and 3′ UTRs

When designing our study, we relied on the findings reported by Berkovits B.D. and Mayr C. [17]. The authors demonstrated that the 3′ untranslated region (3′ UTR) of CD47 plays a critical role in the co-translational localization of CD47 protein [17]. The efficiency of CD47 trafficking to the plasma membrane depends on the 3′ UTR isoform. The long 3′ UTR isoform promotes efficient translocation of CD47 to the cell surface [17]. In contrast, when the short 3′ UTR isoform is used in constructs, CD47 protein predominantly accumulates in the ER [17]. During the synthesis of CD47 protein, HuR and SET proteins bind alternately to its 3′ UTR. Following the synthesis of CD47, it becomes embedded in the ER membrane. RAC1 protein then interacts with the cytoplasmic domain of CD47 and SET, thereby facilitating translocation of CD47 to the plasma membrane [17]. The HuR-binding site (uuuaauagggugagcuugagaguuuucuuucuuucuguuuuuuuuuuuuuuuugacuaauuucacaugcucuaa) in the CD47 3′ UTR increases the protein’s exposure on the cell surface by recruiting specific transport factors [17,18]. It should be noted that translocation of CD47 to the plasma membrane requires both its cytoplasmic tail and HuR-binding site (HuR-BS) [17]. The absence of the HuR-BS or the cytoplasmic tail of CD47 significantly reduces CD47 surface expression [17]. Therefore, in our study, we analyzed the impact of the 5′ and 3′ UTRs of alpha-globin, transferrin, and SARS-CoV-2 on the total expression and the cell surface presentation of the full-length S-protein, S-protein with deletion of the last 19 C-terminal amino acids, as well as S-protein with the last 19 C-terminal amino acid replaced with the cytoplasmic tail of CD47 and the HuR-BS from the CD47 3′ UTR.

The efficiency of S-protein transport to the cell surface is modulated by its cytoplasmic tail. The cytoplasmic tail of S-protein contains 39 amino acids and plays an important role in the intracellular trafficking and translocation of the glycoprotein to the plasma membrane [1,3,7]. It is known that increasing S-protein expression on the cell surface requires the removal of the last 19 C-terminal amino acids [3,6]. This effect is due to a removal of retention and retrieval signals that otherwise limit protein transport to the cell surface.

In our study, we also investigated the impact of replacing the last 19 C-terminal amino acids of the S-protein with the CD47 cytoplasmic tail on S-protein import to the cell surface. We hypothesized that such a chimeric tail would exploit the efficient trafficking machinery of CD47 to enhance plasma membrane localization. The native S-protein (WT), which accumulates in the ERGIC, and its mutant form with a deletion of the last 19 C-terminal amino acids, which is fully transported to the plasma membrane, were used as controls [3]. For these purposes, a set of plasmid constructs was generated, as presented in Figure 2.

Surface S-protein expression was evaluated using a cell-based ELISA, immunofluorescence confocal microscopy, and an analysis of syncytium formation. In all the experiments, native S-protein without stabilization of the prefusion state was used because we planned to analyze the formation of a syncytium. It is known that S-protein in a stabilized prefusion state does not form syncytia [19]. In all the experiments, we used the S-protein without mutations that stabilize the prefusion conformation. In this study, we constructed nine plasmids (Figure 2). Of these, three plasmids encode the full-length S-protein (S-FL) and contain the 5′ and 3′ UTRs of alpha-globin, 5′ and 3′ UTRs of transferrin, or 5′ and 3′ UTRs of SARS-CoV-2. S-FL is the S-protein without any mutations. Another three plasmids encode the S-protein with a deletion of the last 19 C-terminal amino acids (S-dCT19) and containing the 5′ and 3′ UTRs of alpha-globin, transferrin, or SARS-CoV-2. The remaining 3 plasmids encode the S-protein with the last 19 C-terminal amino acids replaced with the cytoplasmic domain of CD47 (S-dCT19/CD47-CT) and containing the 5′ and 3′ UTRs of alpha-globin, transferrin, or SARS-CoV-2, and a HuR-BS in the 3′ UTR (Figure 2).

### 3.2. Introducing the HuR-Binding Site into the 3′ UTR and the Cytoplasmic Tail of CD47 Enhances S-Protein Expression

The SARS-CoV-2 S-protein is a homotrimer. It consists of three chains, each of which forms two subunits: S1 and S2. A furin cleavage site is located between the S1 and S2 subunits. After proteolytic cleavage, the subunits remain bound by hydrogen bonds [20]. The S-protein contains three domains: an ectodomain that binds directly to cellular receptors, a transmembrane domain anchored in the viral membrane, and a short cytoplasmic tail. The ectodomain consists of the receptor-binding S1 subunit and the membrane-bound S2 subunit.

**Figure 3:**
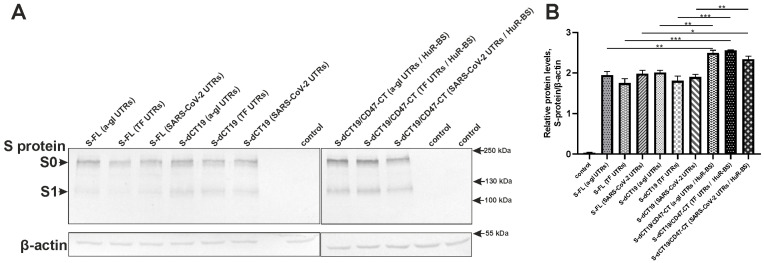
(**A**) Representative Western blot of lysates from HEK293FT-EGFP cells expressing the S-protein. S0—full-length S protein; S1—S1 subunit; control—untransfected HEK293FT-EGFP cells; TF—transferrin; a-gl—alpha-globin. β-actin was used as the loading control. (**B**) Quantification of Western blots; data are presented as the mean ± SD. Differences between groups were assessed using one-way ANOVA followed by Tukey’s HSD test. Significance levels are indicated as * *p* < 0.05, ** *p* < 0.01, and *** *p* < 0.001.

In the current study, we transiently transfected HEK293FT-EGFP with plasmids encoding different S-protein variants containing the 5′ and 3′ UTRs of alpha-globin, transferrin, or SARS-CoV-2 (see Figure 2). S-protein expression was evaluated by Western blotting (Figure 3).

For the Western blot analysis, polyclonal rabbit antibodies against the receptor-binding domain of the S-protein were used. Two major bands were detected at approximately 180 kDa and 110 kDa, corresponding to the full-length S-protein (S0) and the cleaved S1 subunit, respectively (Figure 3). The S1 subunit was detected in all constructs encoding the full-length S-protein (S-FL), as well as the S-dCT19 and S-dCT19/CD47-CT proteins with a modified cytoplasmic tail. Therefore, deletion of the last 19 C-terminal amino acids or inclusion of the CD47 cytoplasmic tail does not affect S-protein cleavage. It is likely that furin-mediated cleavage in the Golgi apparatus still occurs due to the signal peptide and the transmembrane domain, which ensure the correct localization of the S protein.

According to the Western blot results, there were no significant differences in protein expression when the 5′ and 3′ UTRs from alpha-globin, transferrin, or SARS-CoV-2 were introduced into the plasmids in HEK293FT-EGFP cells (Figure 3). However, S-dCT19/CD47-CT constructs containing the cytoplasmic tail of CD47 and the HuR-BS within the 3′ UTR exhibited higher protein levels in HEK293FT-EGFP cells compared to the S-FL and S-dCT19 groups. The study of Berkovits B.D. and Mayr C. demonstrated that the removal of the HuR-BS or cytoplasmic tail of CD47 did not affect the total level of CD47 protein expression but reduced the level of surface protein [17]. We suggest that the observed increase in S-protein expression may be due to a combination of two factors: the replacement of the 19 C-terminal amino acids of the S-protein with the cytoplasmic tail of CD47 and introducing a HuR-BS in the constructs. Since the main goal of our study is to achieve a high level of S-protein presentation on the cell surface, we did not investigate the individual effects of each element (deletion of either the HuR-BS or the cytoplasmic tail of CD47) on total expression and cell surface exposure.

### 3.3. Comparison of Total and Surface S-Protein Levels Through Immunofluorescence Analysis

**Figure 4:**
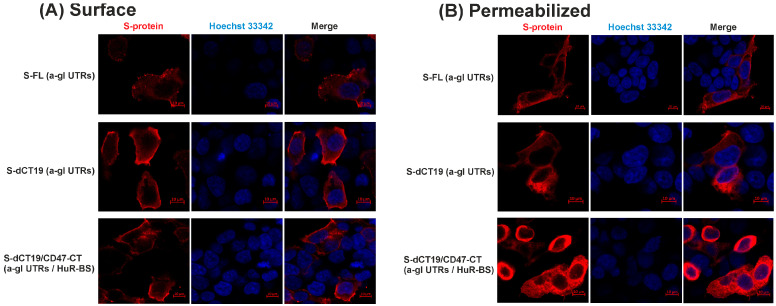
Confocal images of HEK293FT-EGFP cells transiently expressing the SARS-CoV-2 S-protein (WT) and the S-protein with modifications in the cytoplasmic tail. (**A**) HEK293FT-EGFP cells transfected with the indicated constructs. After 36 h, the cells were fixed and stained with an anti-S antibody (red) to detect S-protein localized exclusively to the cell surface. (**B**) HEK293FT-EGFP cells were treated as described in (**A**), but with membrane permeabilization. The Hoechst 33342 dye stains the nuclei (blue). Scale bar represents 10 μm.

Subcellular localization of the S-protein in HEK293FT-EGFP cells was analyzed using confocal immunofluorescence microscopy. In this experiment, we analyzed the S-FL and S-dCT19 constructs containing the 5′ and 3′ UTRs of alpha-globin, as well as S-dCT19/CD47-CT constructs containing the 5′ and 3′ UTRs of alpha-globin and a HuR-BS in the 3′ UTR (Figure 4).

To detect the S-protein on the cell surface, immunofluorescence analysis was performed without cell permeabilization (Figure 4A).

Confocal immunofluorescence microscopy revealed that the full-length S-protein was localized both on the cell surface and intracellularly (Figure 4). Deletion of the last 19 C-terminal amino acids led to increased expression of the protein on the cell surface. After permeabilization, S-FL and S-dCT19 proteins exhibited a cytoplasmic reticular pattern, typical of the endoplasmic reticulum. The S-dCT19/CD47-CT protein, expressed from the S-dCT19/CD47-CT (a-gl UTRs/HuR-BS) construct, was present on the plasma membrane and inside the HEK293FT-EGFP cells. Therefore, introducing the CD47 cytoplasmic tail and the HuR-BS into the 3′ UTR does not block S-protein trafficking to the cell surface.

### 3.4. Cell–Cell Fusion Mediated by the SARS-CoV-2 S-Protein

Syncytium formation (multinuclear cells) is an important mechanism in SARS-CoV-2 pathogenesis, which enables the virus to spread rapidly within tissues [21]. The S-protein on the surface of an infected cell interacts with the ACE2 on neighboring cells, leading to their fusion and the formation of a syncytium. This facilitates viral replication, cell-to-cell spread, and immune evasion [22].

We analyzed syncytium formation to estimate the efficiency with which the S-protein is transported to the cell surface. The efficiency of cell fusion directly depends on the level of S-protein exposure on the cell surface. In addition, the ability of the S-protein to induce cell fusion is an important indicator of its proper maturation [23]. In our study, we used the S-protein without stabilization of the prefusion state. The S-protein does not always react well with antibodies against S-protein, particularly with antibodies against the S1 subunit [24]. This is due to the S-protein existing in a metastable conformation, whereby two S1 subunits of the homotrimer are in a closed conformation, and the third is in an open conformation. In our case, a monoclonal antibody against the S1 subunit did not react with the S-protein. Only a polyclonal antibody against the receptor-binding domain specifically reacted with the S-protein. For this reason, we also evaluated the surface S-protein expression to measure the cell fusion efficiency of the different variants.

**Figure 5:**
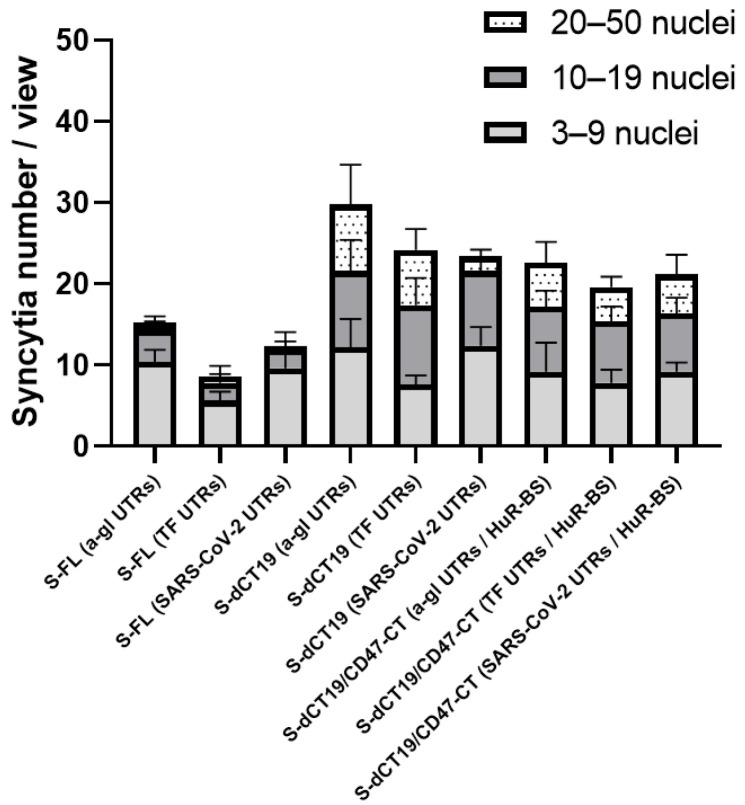
Quantitative analysis of syncytium formation mediated by the S-protein expressed from different plasmids. Data were quantified based on the number of syncytia and nuclei per syncytium. Data are presented as the mean ± SD derived from five images.

We analyzed syncytium formation using constructs that encoded different S-protein variants containing the various 5′ and 3′ UTRs. HEK293FT-EGFP cells expressing different S-protein variants were co-cultured with Vero cells expressing ACE2. While HEK293FT-EGFP cells do not express ACE2, Vero cells exhibit high ACE2 expression and are commonly used to analyze syncytium formation induced by coronaviruses or the S-protein [1]. Syncytium formation was analyzed after 20 h of co-culture (Figure 5 and Supplementary Figures S1 and S2). Cells containing more than three nuclei were identified as a syncytium. The nuclei within a syncytium were grouped together in the center.

Expression of the full-length S-protein from S-FL constructs containing different 5′ and 3′ UTRs resulted in the formation of small syncytia containing 3–9 nuclei on average (Figure 5 and Supplementary Figures S1 and S2). In contrast, deletion of the last 19 C-terminal amino acids of the cytoplasmic domain of the S-protein led to a high level of cell fusion. This was associated with increased S-protein expression on the cell surface compared to the S-FL constructs. Truncation of the last 19 C-terminal amino acids promoted the formation of large syncytia; the number of syncytia containing 10–19 and 20–50 nuclei increased significantly compared to the S-FL constructs. This effect is explained by the removal of the ER retrieval signal [3].

The S-dCT19/CD47-CT constructs induced syncytium formation, similar to the S-dCT19 constructs (Figure 5 and Supplementary Figures S1 and S2). The fusogenic activity of the S-dCT19 and S-dCT19/CD47-CT constructs was comparable. Furthermore, these constructs demonstrated significantly higher fusogenic activity than the S-FL constructs.

**Figure 6:**
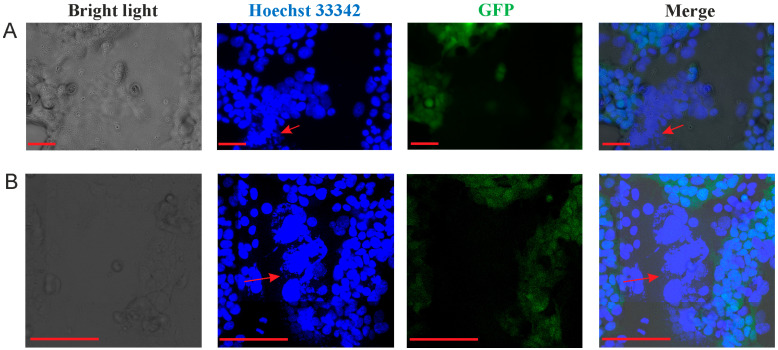
Syncytia with fragmented nuclei. (**A**) Fluorescence microscopy; scale bar length corresponds to 50 μm. (**B**) Confocal fluorescence microscopy; scale bar length corresponds to 100 μm. Fragmented nuclei are indicated by the red arrows.

In addition, we observed syncytia containing fragmented nuclei (Figure 6). The formation of virus-induced syncytia is a well-known pathogenic mechanism [21]. SARS-CoV-2-mediated syncytia are destroyed, resulting in cell death at later stages. It has been established that syncytia induced by coronaviruses can undergo apoptosis and pyroptosis [21,25]. Lee J. et al. demonstrated that S-protein-mediated cell fusion triggers TP53 stabilization, alterations in chromatin accessibility, and senescence activation [26]. The extent of these effects varies according to the fusogenicity of different SARS-CoV-2 spike protein variants. Therefore, the cytotoxicity of the constructs was tested.

### 3.5. Comparison of Total and Cell Surface S-Protein Expression Through Cell-Based ELISA Analysis

Cell-based enzyme-linked immunosorbent assay (cell-based ELISA) is an immunoassay method used for the rapid detection of antigens or receptors expressed on the cell surface [27]. It is used for hybridoma screening to produce monoclonal antibodies, as well as for the analysis of various cell surface and intracellular antigens. This assay was used to analyze anti-SARS-CoV-2 antibodies isolated from convalescent patients using SARS-CoV-2-infected Vero cells [28].

In this study, we used a cell-based ELISA to detect the S-protein. Vero cells transiently expressing the S-protein were fixed and used to perform cell-based ELISA (described in Section 2.4). To analyze surface S-protein levels, the cells were not permeabilized. Total S-protein (internal and surface) levels were analyzed after cell permeabilization. However, it should be noted that cell permeabilization with 0.05–0.1% Triton X-100 leads to a partial loss of surface antigens despite fixation [29]. Therefore, in this experiment, when determining total protein expression after cell permeabilization, we predominantly detected the internal protein level and some surface proteins.

**Figure 7:**
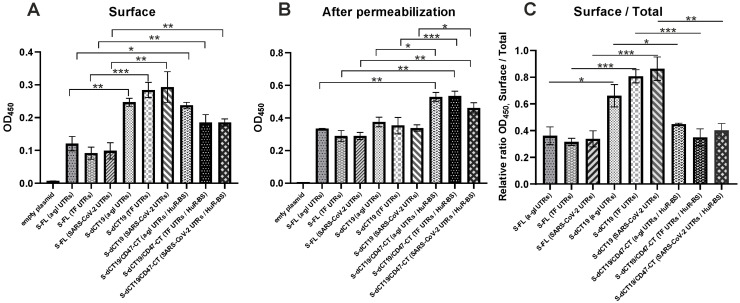
Comparison of cell surface (without permeabilization) (**A**) and total (after permeabilization) (**B**) S-protein expression in Vero cells using cell-based ELISA. (**C**) The OD_450_ ratio between surface and total S-protein levels. Data are presented as the mean ± SD. Differences between groups were assessed using one-way ANOVA followed by Tukey’s HSD test. Significance levels are indicated as * *p* < 0.05, ** *p* < 0.01, and *** *p* < 0.001.

According to the obtained data, the full-length S-protein expressed from the S-FL constructs was localized both inside and on the cell surface (Figure 7 and Supplementary Figure S3).

After cell permeabilization, the level of S-FL protein significantly increased (Figure 7B and Supplementary Figure S3). No difference in S-FL expression was observed when the 5′ and 3′ UTRs of alpha-globin, transferrin, or SARS-CoV-2 were included in the constructs.

Deletion of the last 19 C-terminal amino acids of the S-protein cytoplasmic tail significantly increased S-protein cell surface presentation compared to the S-FL constructs (Figure 7A and Supplementary Figure S3). This suggests that the S-protein cytoplasmic tail plays a key role in intracellular retention. However, no significant differences were observed when different 5′ and 3′ UTRs were introduced into the plasmids encoding the S-protein with a truncated cytoplasmic tail.

The most significant effect on the expression levels was achieved with S-dCT19/CD47-CT constructs that contained the CD47 cytoplasmic tail and the HuR-BS in the 3′ UTR. After permeabilization of cells transfected with S-dCT19/CD47-CT constructs, a significantly higher level of protein expression was observed compared to the S-FL and S-dCT19 constructs (Figure 7B and Supplementary Figure S3). The introduction of the HuR-BS into the 3′ UTRs and the cytoplasmic tail of CD47 significantly enhanced both the total expression and the cell surface presentation of the S-dCT19/CD47-CT protein with all the tested 5′ and 3′ UTRs (alpha-globin, transferrin, and SARS-CoV-2) compared to the constructs encoding the S-FL protein with the same UTRs (Figure 7 and Supplementary Figure S3). It should be noted that we did not observe any statistically significant differences in the surface levels of the S-protein expressed from the S-dCT19 and S-dCT19/CD47-CT constructs (Figure 7A). However, the total protein level expressed from the S-dCT19/CD47-CT constructs was significantly higher than that of the S-dCT19 constructs.

Thus, introducing the 5′ and 3′ UTRs of alpha-globin, transferrin, or SARS-CoV-2 alone did not have a significant impact on the total expression level of S-protein from the S-FL and S-dCT19 constructs. The greatest effect on the total expression was achieved by introducing the cytoplasmic tail of CD47 and a HuR-BS into the 3′ UTR, rather than simply replacing the 5′ and 3′ UTRs.

It should be noted that the total S-protein level quantified by cell-based ELISA (after permeabilization) correlated with the data obtained from the Western blot analysis (Figure 3). The S-dCT19/CD47-CT constructs demonstrated elevated total S-protein expression compared to both the S-FL and S-dCT19 constructs. To evaluate the efficiency of S-protein translocation to the cell surface, the surface presentation efficiency was calculated for all constructs, which was defined as the ratio of surface S-protein to total S-protein (Figure 7C). According to this calculation, the introduction of the cytoplasmic tail of CD47 and the HuR-BS into the 3′ UTR did not affect the efficiency of S-protein presentation on the cell surface, which remained similar to that observed for the S-FL constructs (Figure 7C). The observed increase in the amount of S-protein expressed from the S-dCT19/CD47-CT constructs on the cell surface, compared to the S-FL constructs, was associated with an increase in the overall expression level (Figure 7A,C). Therefore, introducing the cytoplasmic tail of CD47 and the HuR-BS into the 3′ UTR does not block S-protein trafficking to the plasma membrane. However, the S-dCT19/CD47-CT constructs did not enhance S-protein transport to the cell surface compared to the S-dCT19 constructs. Importantly, this modification results in a significant increase in total S-protein expression compared to the S-FL and S-dCT19 constructs. These findings identify the S-dCT19/CD47-CT constructs as promising candidates due to their capacity to support high-level production of the S-protein.

### 3.6. Cytotoxicity Analysis of Constructs Encoding Different Variants of the S-Protein

The cytotoxicity of the S-protein-encoding constructs was analyzed in HEK293 and Vero cells. HEK293 cells do not express ACE2, whereas Vero cells exhibit high levels of ACE2 expression. This difference enabled us to evaluate any additional cytopathic effects that might be mediated by the interaction between the S-protein and the receptor and the subsequent syncytium formation. Therefore, we investigated the impact of the different plasmid constructs in both cell types.

**Figure 8:**
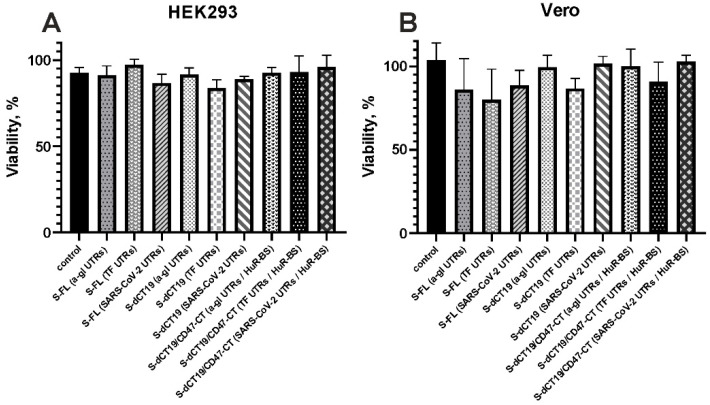
The viability of cells treated by different plasmid constructs. (**A**) The viability of HEK293 cells; (**B**) the viability of Vero cells. The cell survival rates of nine samples were compared. Statistical agreement was observed across all samples, including the control samples.

The MTT assay results demonstrated that none of the tested constructs caused significant cytotoxicity in either HEK293 or Vero cells (Figure 8).

Although the S-dCT19 and S-dCT19/CD47-CT constructs induced high levels of syncytium formation, this did not result in a statistically significant decrease in Vero cell viability. Syncytium formation can be pathological, leading to cell death [27]. The absence of the expected cytopathic effect mediated by cell fusion may be explained by the low basal expression of the proteases required for efficient S-protein cleavage and activation of the S-protein, such as furin and TMPRSS2, in Vero cells compared to HEK293 cells [30].

Thus, the obtained data indicate that even high levels of both surface and total spike protein expression in the model system used do not lead to significant toxicity. This is a favorable characteristic for their potential application.

## 4. Discussion

Recently, there has been much attention given to the localization of S-protein in the cell since understanding the mechanisms of cellular protein transport is important for the development of vaccines. As mentioned above, the S-protein predominantly accumulates in intracellular compartments during biosynthesis [1,2]. In the context of mRNA- or adenovirus-based vaccines encoding the S-protein, only antigens on the cell surface are recognized by B-cell receptors to trigger B-cell activation and subsequent antibody production [9]. Therefore, for the development of highly effective vaccines, high antigen translation is insufficient. High antigen expression on the cell surface is also necessary. High expression of the S-protein on the cell surface may lead to a reduction in the required vaccine dose and, consequently, reduced side effects from vaccination.

In our study, we specifically modified genetic constructs encoding the SARS-CoV-2 S-protein to increase its total expression and cell surface presentation. Our data robustly demonstrate that the cytoplasmic tail is a key factor limiting the surface expression of the full-length S-protein. We confirmed that deletion of the last 19 C-terminal amino acids containing the ER retrieval signal significantly increased the S-protein abundance on the cell surface. This enhanced its fusogenic activity and led to the formation of large syncytia. These findings are consistent with published data on the involvement of the S-protein cytoplasmic tail in cellular trafficking and syncytium formation [1,3].

The development of the optimal mRNA vaccine requires balancing mRNA stability, translation efficiency, and cell surface antigen presentation while minimizing cytotoxic effects both at the cellular and organismal levels. The 5′ and 3′ UTRs of human alpha-globin mRNA are highly GC-rich and thus stable, resulting in high levels of protein translation [15]. Therefore, in this study, we selected not only the alpha-globin UTRs, but also the SARS-CoV-2 UTRs and the human transferrin UTRs. However, we observed no significant differences in protein expression levels in cells following transfection with plasmid constructs containing the 5′ and 3′ UTRs from alpha-globin, transferrin, or SARS-CoV-2.

Therefore, in the present study, we also introduced the HuR-BS from the CD47 3′ UTR at the end of the 3′ UTR and the cytoplasmic tail of CD47 in plasmid constructs encoding S-protein (S-dCT19/CD47-CT constructs). We hoped that these modifications would enhance S-protein expression and promote S-protein trafficking to the cell surface. The S-dCT19/CD47-CT constructs in which the 19 C-terminal amino acids of the S-protein were replaced with the cytoplasmic tail of CD47, and a HuR-BS was introduced into the 3′ UTRs of alpha-globin, transferrin, and SARS-CoV-2, demonstrated high levels of protein expression compared to the S-FL and S-dCT19 constructs. These constructs also demonstrated high fusogenic activity, comparable to the S-dCT19 constructs, indicating efficient transport of the chimeric protein to the cell surface. Comparable levels of S-protein were observed on the cell surface for the S-dCT19 and S-dCT19/CD47-CT constructs. In contrast to the S-dCT19 constructs, a significant increase in total S-protein expression was observed for the S-dCT19/CD47-CT constructs. However, the final efficiency of surface presentation of the S-protein expressed from the S-dCT19/CD47-CT constructs was no different from that of the S-FL constructs (Figure 7C). We attribute this to the fact that by introducing of the CD47 cytoplasmic tail, we anchor the S-protein to the ER membrane again [17,18]. This once again confirms that deletion of the 19 C-terminal amino acids of the S-protein is important for protein translocation to the cell surface.

When introducing the CD47 cytoplasmic tail and the HuR-BS into the 3′ UTR for proteins other than CD47 or S-protein, it should be noted that high protein expression could be toxic. In our study, no toxicity was observed with any constructs according to MTT assay. Overall, we believe that the S-dCT19/CD47-CT constructs are promising due to their ability to provide high S-protein expression. These constructs have the potential to be used as templates for mRNA vaccine synthesis. However, to develop a safe vaccine construct, it is necessary to introduce at least the K986P-V987P mutations into the S-protein to stabilize its prefusion conformation, thereby reduce the likelihood of pathological syncytium formation.

### Limitations

The results of this study cannot be applied directly to the design of mRNA vaccines.

In this study, we used plasmid constructs encoding the S-protein without a stabilized prefusion state since we analyzed S-protein-mediated syncytium formation. While this allowed us to study the fusogenic activity, the current standard for creating safe and effective vaccines against SARS-CoV-2 is the stabilized prefusion form of the S-protein (specifically, Spike-2P or Spike-6P) as this directs the immune response towards neutralization-sensitive epitopes while minimizing the pathological effects associated with syncytium formation.

Furthermore, this study was conducted using plasmid constructs whose transcription occurs in the nucleus, unlike mRNA vaccines, which function in the cell cytoplasm. This key difference may affect parameters such as mRNA stability, translation efficiency, and immunogenicity. As the plasmids obtained in this study are intended for potential use in mRNA vaccines, it is necessary to confirm that the identified advantages in protein expression are retained when switching to an in vitro transcribed mRNA (IVT-RNA) platform.

## 5. Conclusions

The cellular localization of the S-protein can influence how efficiently it is recognized as a foreign antigen and processed by the immune system to generate an immune response. Rational distribution of the antigen within the cell, in this case, the SARS-CoV-2 S-protein, is likely to reduce the required vaccine dose, thereby minimizing some of the side effects. In this study, we attempted to a construct plasmid that would increase the total level and cell surface presentation of the SARS-CoV-2 S-protein. We designed nine plasmid constructs encoding the S-protein with different modifications to its cytoplasmic tail and containing the 5′ and 3′ UTRs of alpha-globin, transferrin, or SARS-CoV-2 (Figure 2). The replacement of the 5′ and 3′ UTRs did not have a significant effect on S-protein expression levels. We confirmed that the cytoplasmic tail of the S-protein plays a key role in limiting its localization to the plasma membrane. Deletion of the last 19 C-terminal amino acids, which contain the ER retrieval signal, significantly increased S-protein presentation on the cell surface and enhanced its fusogenic activity. Introducing the HuR-BS from the CD47 3′ UTR into the constructs’ 3′ UTR and replacement of the C-terminal region of the S-protein with the cytoplasmic tail of CD47 significantly increased the total level of the S-protein compared to the S-FL and S-dCT19 constructs. The S-dCT19/CD47-CT constructs did not block the transport of S-protein to the cell surface and did not enhance S-protein trafficking to the cell surface compared to the S-dCT19 constructs. The high fusogenic activity of the S-dCT19/CD47-CT constructs, which was comparable to that of S-dCT19, confirmed successful localization of the chimeric protein on the cell surface. Although the S-dCT19/CD47-CT constructs demonstrated higher presentation of the S-protein on the cell surface than the S-FL constructs, this difference was associated with higher total S-protein expression. Overall, the S-dCT19/CD47-CT constructs are promising due to their high S-protein expression.

## Data Availability

Data is contained within the article or Supplementary Materials.

## Supplementary Materials

The following supporting information can be downloaded at: https://www.mdpi.com/article/10.3390/v18010137/s1. Supplementary File 1: Table S1—nucleotide sequences of 5′ and 3′ UTRs, HuR-binding site; Table S2—list of oligonucleotides used for introducing the 19 C-terminal amino acids of the cytoplasmic domain; Figure S1—syncytium formation assay for the full-length SARS-CoV-2 S-protein (S-FL), the S-protein with mutations in the cytoplasmic domain (S-dCT19, S-dCT19/CD47-CT), and with various 5′ and 3′ UTR; Figure S2—comparison of syncytium formation mediated by S-protein from different plasmids.; Figure S3—comparison of cell surface and total S-protein expression by cell-based ELISA in non-permeabilized and permeabilized Vero cells. Supplementary File 2: original images for Western blotting and immunofluorescence confocal microscopy.

## Abbreviations

The following abbreviations are used in this manuscript:

ACE2	angiotensin-converting enzyme 2
ER	endoplasmic reticulum
ERGIC	the intermediate compartment between the endoplasmic reticulum and Golgi apparatus
HuR	human antigen R
HuR-BS	HuR-binding site
5′ and 3′ UTRs	5′ and 3′ untranslated regions
