# Supplementary material for: The Introduction of a HuR-Binding Site in the 3′ UTR and the CD47 Cytoplasmic Tail Enhances SARS-CoV-2 S-Protein Expression in Cells
Source: Viruses. 2026 Jan 21;18(1):137. doi: 10.3390/v18010137 (PMC12846518; doi:10.3390/v18010137)

Table S1. Nucleotide sequences of 5′ and 3′ UTRs, HuR-binding site

5′ or 3′ UTRs	Source	Sequence	Oligonucleotides
5′ UTR of human alpha globin	NG_000006	ACTCTTCTGGTCCCCACAGACTCAGAGAGAACCC	5′-**P**-GACTCAGAGAGAACCCCTCGAGGCCACCATGTTCGTTTTCCTTGTTCTGTTG-3′,<br>5′-**P**-TGTGGGGACCAGAAGAGTAAGCTTAATTCTGACGGTTCACTAAACG-3′
5′ UTR of SARS-CoV-2	NC_045512.2	ATTAAAGGTTTATACCTTCCCAGGTAACAAACCAACCAACTTTCGA TCTCTTGTAGATCTGTTCTCTAAACGA	5′-TTTTTTAAGCTTATTAAAGGTTTATACCTTCCCAGGTAACAAACCAACCAACTTTCGA-3′<br>5′-TTTTTTCTCGAGTCGTTTAGAGAACAGATCTACAAGAGATCGAAAGTTGGTTGGTTTGT-3′
5′ UTR of human transferrin	NG_013080	ACAGAAGCGAGTCCGACTGTGCTCGCTGCTCAGCGCCGCACCCGG	5′-TTTTTTAAGCTTACAGAAGCGAGTCCGACTGTGCTCGCTGCTCAGCGCCG-3′<br>5′-TTTTTTCTCGAGCCGGGTGCGGCGCTGAGCAGCG-3
3′ UTR of human alpha-globin	NG_000006	GCTGGAGCCTCGGTGGCCATGCTTCTTGCCCCTTGGGCCTCCCCCC AGCCCCTCCTCCCCTTCCTGCACCCGTACCCCCGTGGTCTTTGAATA AAGTCTGAGTGGGCGGCA	3 ′ UTR of alpha-globin was generated by annealing four DNA oligonucleotides, and then cloned into plasmid pUC19.<br>Oligonucleotides for assembly:<br>Olig 1 GCTGGAGCCTCGGTGGCCATGCTTCTTGCCCCTTGGGCCTCCCCCC<br>Olig 2 GGGGCTGGGGGGAGGCCCA<br>Olig 3 CCCCCCAGCCCCTCCTCCCCTTCCTGCACCCGTACCCCCG<br>Olig 4 TGCCGCCCACTCAGACTTTATTCAAAGACCACGGGGGTACGGGTG<br><br>Primers for the introduction of XbaI and EcoRV sites in 3′ UTR of alpha-globin, for subsequent cloning into a S-dCT19 (alpha-globin UTRs) plasmid:<br>Forward primer ATATTAGATATCGCTGGAGCCTCGGTGGCC<br>Reverse primer TCCTATTCTAGATGCCGCCCACTCAGACTTTATTC
3′ UTR of SARS-CoV-2	NC_045512.2	ACTCATGCAGACCACACAAGGCAGATGGGCTATATAAACGTTTTC GCTTTTCCGTTTACGATATATAGTCTACTCTTGTGCAGAATGAATTC TCGTAACTACATAGCACAAGTAGATGTAGTTAACTTTAATCTCACA TAGCAATCTTTAATCAGTGTGTAACATTAGGGAGGACTTGAAAGA GCCACCACATTTTCACCGAGGCCACGCGGAGTACGATCGAGTGTA CAGTGAACAATGCTAGGGAGAGCTGCCTATATGGAAGAGCCCTAA TGTGTAAAATTAATTTTAGTAGTGCTATCCCCATGTGATTTTAATA GCTTCTTAGGAGAATGAC	3 ′ UTR of SARS-CoV-2 was synthetically assembled using 10 oligonucleotides, and then cloned into plasmid pUC19.<br>Oligonucleotides for assembly:<br>Olig 1 ACTCATGCAGACCACACAAGGCAGATGGGCTATATAAACGTTTTCGCTTTTCCGT<br>Olig 2 ATTCATTCTGCACAAGAGTAGACTATATATCGTAAACGGAAAAGCGAAAACGT<br>Olig 3 GTCTACTCTTGTGCAGAATGAATTCTCGTAACTACATAGCACAAGTAGATGTAGT<br>Olig 4 ATTAAAGATTGCTATGTGAGATTAAAGTTAACTACATCTACTTGTGCTATGTAGT<br>Olig 5 AACTTTAATCTCACATAGCAATCTTTAATCAGTGTGTAACATTAGGGAGGACTTG<br>Olig 6 GCGTGGCCTCGGTGAAAATGTGGTGGCTCTTTCAAGTCCTCCCTAATGTTACACA<br>Olig 7 CACCGAGGCCACGCGGAGTACGATCGAGTGTACAGTGAACAATGCTAGGGAG<br>Olig 8 ATTTTACACATTAGGGTTCTTCCATATAGGCAGCTCTCCCTAGCATTGTTCACTG<br>Olig 9 ATGGAAGAACCCTAATGTGTAAAATTAATTTTAGTAGTGCTATCCCCATGTG<br>Olig 10 GTCATTCTCCTAAGAAGCTATTAAAATCACATGGGGATAGCACTACT<br><br>Primers for the introduction of XbaI and EcoRV sites in 3′ UTR of SARS-CoV-2, for subsequent cloning into a S-dCT19 (SARS-CoV-2 UTRs) plasmid:<br>Forward primer ATATTAGATATCACTCATGCAGACCACACAAGGC<br>Reverse primer TCCTATTCTAGAGTCATTCTCCTAAGAAGCTATTAAAATCACATG
3′ UTR of human transferrin	NG_013080	AATCTCAGAGGTAGGGCTGCCACCAAGGTGAAGATGGGAACGCAG ATGATCCATGAGTTTGCCCTGGTTTCACTGGCCCAAGTGGTTTGTG CTAACCACGTCTGTCTTCACAGCTCTGTGTTGCCATGTGTGCTGAA CAAAAAATAAAAATTATTATTGATTTTATATTTCAAAAACTCCATT CTTTCCTAAATATTTTCAACAAAGGATTTCTTTATGCATTCTGCCTA AATACCTATGCAACTGAGCCCTTCCTTCTCAGCTCAAGATTCGTCT GGTCTTTCCCTACAGCTTTGTGTGTGCCATGGCCACATCTCCTGGGT ACAGTTCAAGGAGACATCTTTTCTAAAAGGGTCTGCGTGATCATTA AAATATAATCAAATGTA	3 ′ UTR of transferrin was synthetically assembled using 10 oligonucleotides, and then cloned into plasmid pUC19.<br>Oligonucleotides for assembly:<br>Olig 1 AATCTCAGAGGTAGGGCTGCCACCAAGGTGAAGATGGGAACGCAGATGATCCATGAGT<br>Olig 2 ACGTGGTTAGCACAAACCACTTGGGCCAGTGAAACCAGGGCAAACTCATGGATCATCTGCGT<br>Olig 3 GGTTTGTGCTAACCACGTCTGTCTTCACAGCTCTGTGTTGCCATGTGTGCTGAACA<br>Olig 4 AATGGAGTTTTTGAAATATAAAATCAATAATAATTTTTATTTTTTGTTCAGCACACATGGCA<br>Olig 5 AAAATTATTATTGATTTTATATTTCAAAAACTCCATTCTTTCCTAAATATTTTCAACAAAG<br>Olig 6 GCATAGGTATTTAGGCAGAATGCATAAAGAAATCCTTTGTTGAAAATATTTAGGAAAGAATGG<br>Olig 7 GCATTCTGCCTAAATACCTATGCAACTGAGCCCTTCCTTCTCAGCTCAAGATTCGTCTGG<br>Olig 8 CCCAGGAGATGTGGCCATGGCACACACAAAGCTGTAGGGAAAGACCAGACGAATCTTGAGCTG<br>Olig 9 GGCCACATCTCCTGGGTACAGTTCAAGGAGACATCTTTTCTAAAAGGGTCTGCGTGATCA<br>Olig 10 TACATTTGATTATATTTTAATGATCACGCAGACCCTT<br><br>Primers for the introduction of BamHI site in 3′ UTR of transferrin, for subsequent cloning into a pCMV14-3X-Flag-SARS-CoV-2:<br>Forward primer ATATTAGGATCCTGATAAGATATCAATCTCAGAGGTAGGGCTGCCAC<br>Reverse primer TCCTATTCTAGAGTCATTCTCCTAAGAAGCTATTAAAATCACATG<br>We have selected colonies containing the sequence in the correct orientation.

HuR-binding site (HuR-BS) from 3´ UTR of CD47	NM_000734	TTTAATAGGGTGAGCTTGAGAGTTTTCTTTCTTTCTGTTTTTTTTTTT<br>TTTTTGACTAATTTCACATGCTCTAA	Primers for the introduction of HuR-BS in 3´ UTR alpha globin:<br><br>Forward primer<br>AAAGGTATGATGAATGACGAGTGATAAGATATCGCTGGAGCCTCGGTGGCC<br>Reverse primer<br>TTAGAGCATGTGAAATTAGTCAAAAAAAAAAAAAAAAACAGAAAGAAAGAAAACTCTC<br>AAGCTCACCCTATTAAATGCCGCCCACTCAGACTTTATTC<br><br>Primers for the introduction of HuR-BS in 3´ UTR transferrin:<br>Forward primer<br>AAAGGTATGATGAATGACGAGTGATAAGATATCAATCTCAGAGGTAGGGCTGCCAC<br>Reverse primer<br>TTAGAGCATGTGAAATTAGTCAAAAAAAAAAAAAAAAACAGAAAGAAAGAAAACTCTC<br>AAGCTCACCCTATTAAATACATTTGATTATATTTTAATGATCACGCAGAC<br><br>Primers for the introduction of HuR-BS in 3´ UTR SARS-CoV-2:<br>Forward primer<br>AAAGGTATGATGAATGACGAGTGATAAGATATCACTCATGCAGACCACACAAGGC<br>Reverse primer<br>TTAGAGCATGTGAAATTAGTCAAAAAAAAAAAAAAAAACAGAAAGAAAGAAAACTCTC<br>AAGCTCACCCTATTAAAGTCATTCTCCTAAGAAGCTATTAAAATCACATG
Cytoplasmic tail of CD47	NM_000734	AAATTTGTGGCTTCCAATCAGAAGACTATACAAC	Olig 1 GGATCCAAATTTGTCGCTTCTAACCAGAAAAC<br>Olig 2 CCTGGGGGGTTGAATAGTTTTCTGGTTAGAAGCGAC<br>Olig 3 ATTCAACCCCCCAGGAAAGCGGTCGAAGAGCC<br>Olig 4 TCTTTAAAGGCATTCAGGGGCTCTTCGACCGC<br>Olig 5 CCCCTGAATGCCTTTAAAGAATCTAAAGGTATGATGAATGACGAG<br>Olig 6 GATATCTTATCACTCGTCATTCATCATACCTT

Table S2. List of oligonucleotides used for introducing the 19 C-terminal amino acids of the cytoplasmic domain

Sequence/mutation	Oligonucleotides
19 C-terminal AA residues of the cytoplasmic domain of the SARS-CoV-2 S protein (AAATTTGATGAAGATGACAGTGAACCAGTCCTGAAAG GGGTCAAGCTGCATTACACA)	Primers modified with a phosphate group at the 5´ position were used:<br><br>S-dCT19 (a-gl UTRs) construct:<br>Forward primer 5´-P-GGTCAAGCTGCATTACACATGATAAGATATCGCTGGAGCCTCGG-3´<br>Reverse primer 5´-P-CCTTTCAGGACTGGTTCACTGTCATCTTCATCAAATTTGCAACATGATCCGCAAGAGCAGC-3´<br><br>S-dCT19 (TF UTRs) construct:<br>Forward primer 5´-P-GGTCAAGCTGCATTACACATGATAAGATATCAATCTCAGAGGTAGGGCTG-3´,<br>Reverse primer 5´-P-CCTTTCAGGACTGGTTCACTGTCATCTTCATCAAATTTGCAACATGATCCGCAAGAGCAGC-3´<br><br>S-dCT19 (SARS-CoV-2 UTRs) construct:<br>Forward primer 5´-P- GGTCAAGCTGCATTACACATGATAAGATATCACTCATGCAGACCACACA-3´,<br>Reverse primer 5´-P-CCTTTCAGGACTGGTTCACTGTCATCTTCATCAAATTTGCAACATGATCCGCAAGAGCAGC-3´

Figure S1.

Syncytium formation assay for the full-length SARS-CoV-2 S-protein (S-FL), the S-protein with mutations in the cytoplasmic domain (S-dCT19, S-dCT19/CD47-CT), and with various 5´ and 3´ UTR. Vero cells were co-cultured with HEK293FT-EGFP cells expressing either S protein. After 20 h of co-culture, syncytia were analyzed. Cells were fixed in 4% paraformaldehyde and nuclei were stained with Hoechst 33342. A red arrowhead indicates a syncytium. The scale bar represents 100 μm.

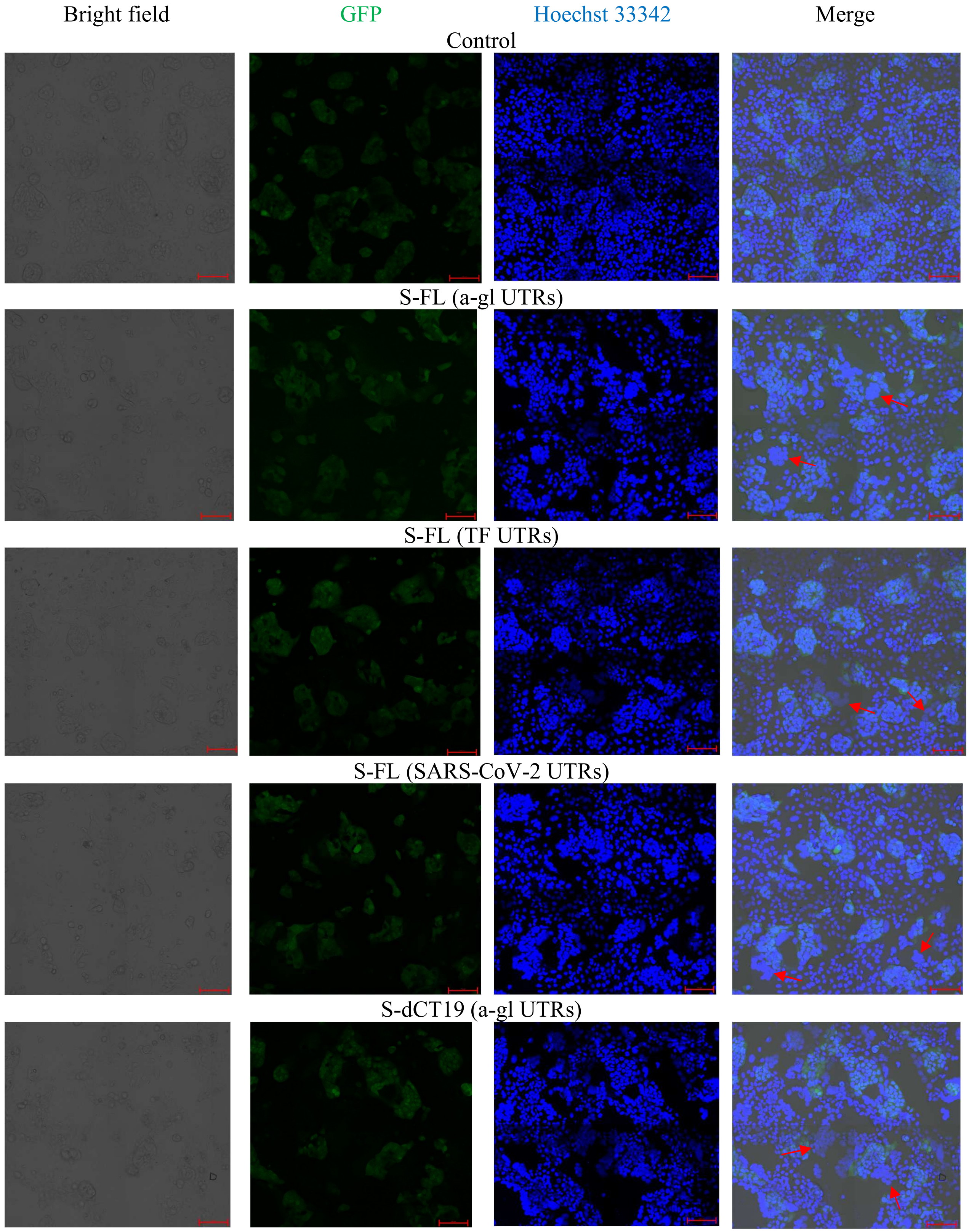

Figure S1 (continued)

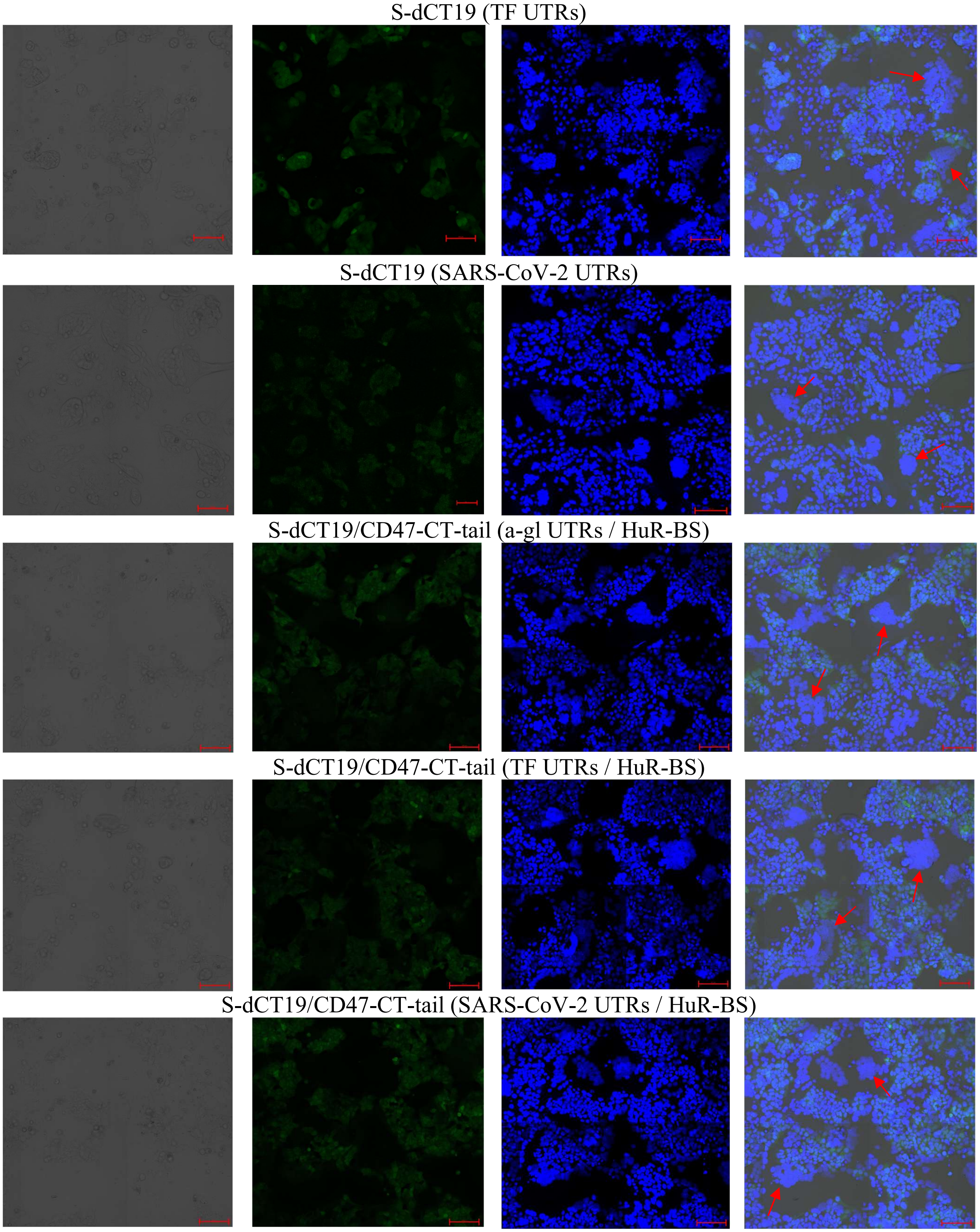

Figure S2. Comparison of syncytium formation mediated by S-protein from different plasmids. Box plots show syncytia numbers per view, stratified by nucleus count: syncytia with 3–9 nuclei (panel A), syncytia with 10–19 nuclei (panel B), syncytia with 20–50 nuclei (panel C). Central line means median; box boundaries mean 25th–75th percentiles; whiskers mean 5th–95th percentiles

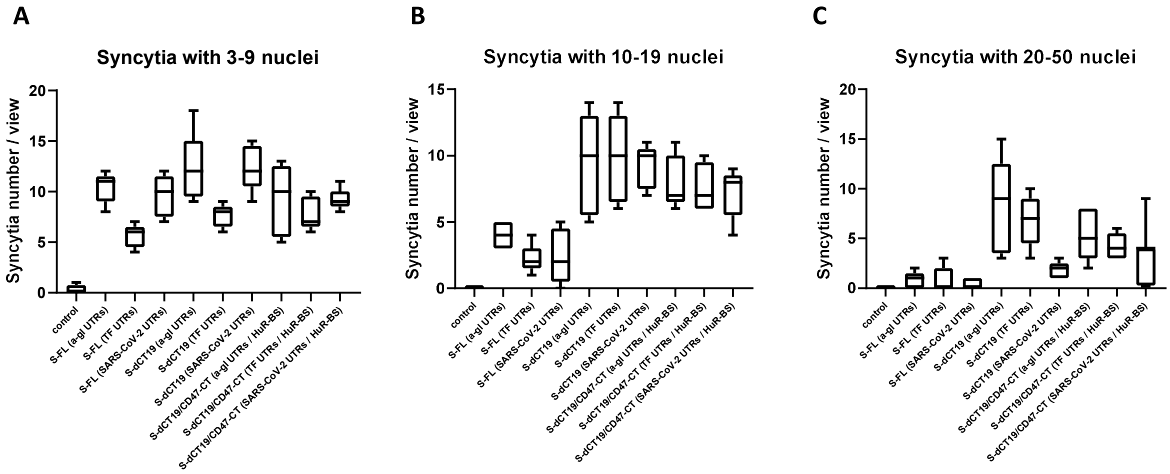

Figure S3. Comparison of cell surface and total S-protein expression by cell-based ELISA in non-permeabilized (A) and permeabilized (B) Vero cells, respectively. Data are presented as the mean ± SD. Statistical significance was analyzed by the two-tailed Student's t-test. $P < 0.05$ was considered as statistical significance. Ns indicates $P > 0.05$; * indicates $P < 0.05$.

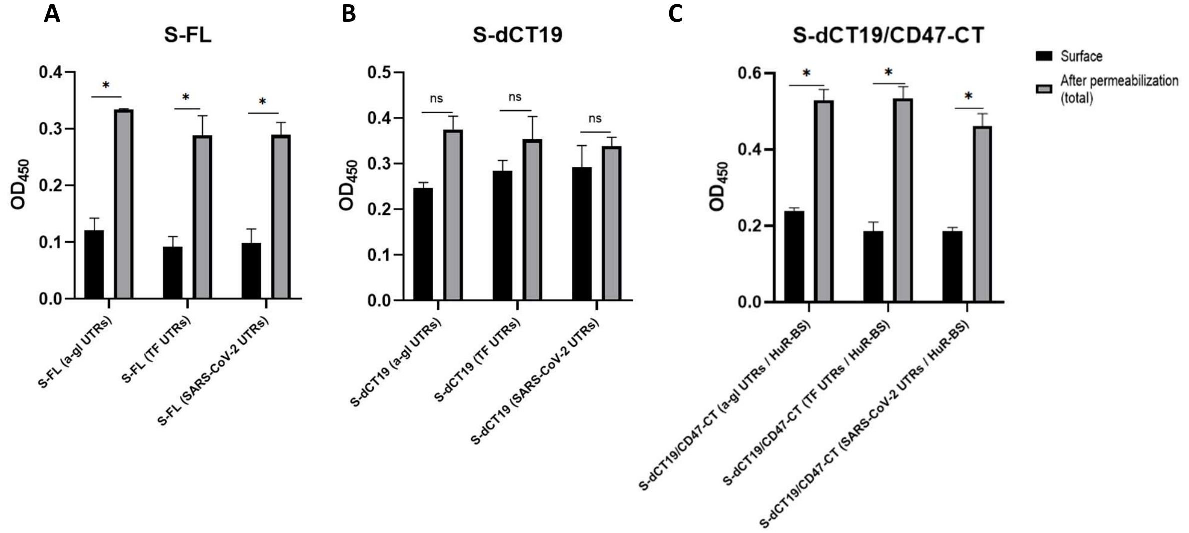

Supplement: Supplementary file 1 [file viruses-18-00137-s001.zip › Supplementary file 1.pdf]
