# Supplementary material for: The Introduction of a HuR-Binding Site in the 3′ UTR and the CD47 Cytoplasmic Tail Enhances SARS-CoV-2 S-Protein Expression in Cells
Source: Viruses. 2026 Jan 21;18(1):137. doi: 10.3390/v18010137 (PMC12846518; doi:10.3390/v18010137)
Supplement: Supplementary file 1 [file viruses-18-00137-s001.zip › Supplementary file 2 (original images)/Western Blot/Western Blot.pdf]

Original images of Western blots

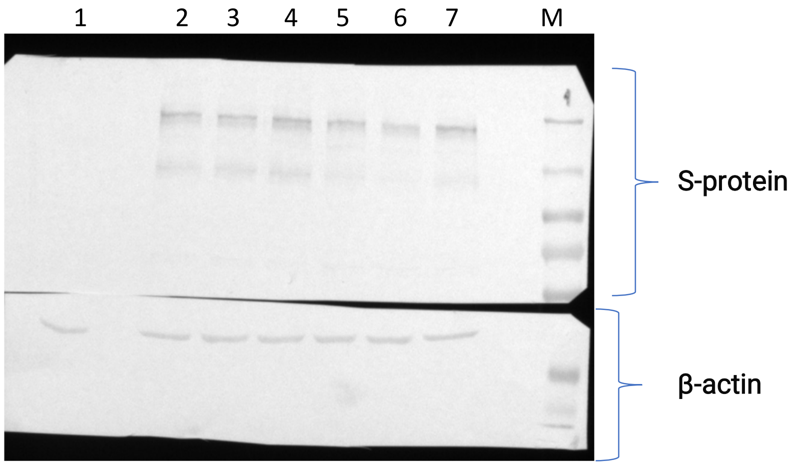

Gel 1 / PVDFmembrane 1. Representative Western blot of lysates from HEK293FT-GFP cells expressing the S-protein.
1 – Control (untransfected HEK293FT-GFP cell); **2** – S-dCT19 (SARS-CoV-2 UTRs) construct; **3** – S-dCT19 (TF UTRs) construct; **4** – S-dCT19 (a-gl UTRs) construct; **5** – S-FL (SARS-CoV-2 UTRs) construct; **6** – S-FL (TF UTRs) construct; **7** – S-FL (a-gl UTRs) construct; **M** – Prestained Protein Ladder 10–250 kDa (G26619-250UL, Servicebio). TF – transferrin, a-gl – alpha-globin.

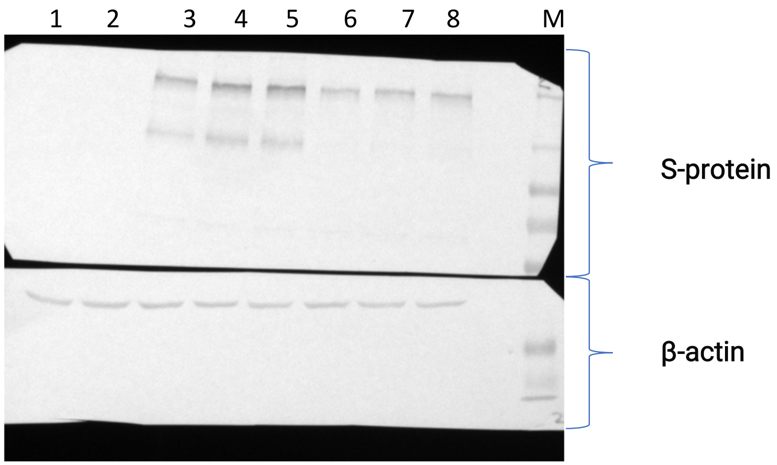

Gel 2 / PVDFmembrane 2. Representative Western blot of lysates from HEK293FT-GFP cells expressing the S-protein.
1 – Control (untransfected HEK293FT-GFP cell); **2** – Control (untransfected HEK293FT-GFP cell); **3** – S-dCT19/CD47-CT (SARS-CoV-2 UTRs / HuR-BS) construct; **4** – S-dCT19/CD47-CT (TF UTR / HuR-BS) construct; **5** – S-dCT19/CD47-CT (a-gl UTR / HuR-BS) construct; **6** – S-FL (SARS-CoV-2 UTRs) construct; **7** – S-FL (TF UTRs) construct; **8** – S-FL (a-gl UTRs) construct; **M** – Prestained Protein Ladder 10–250 kDa (G26619-250UL, Servicebio). TF – transferrin, a-gl – alpha-globin.
