# Supplementary figures and images for: The Introduction of a HuR-Binding Site in the 3′ UTR and the CD47 Cytoplasmic Tail Enhances SARS-CoV-2 S-Protein Expression in Cells
Source: Viruses. 2026 Jan 21;18(1):137. doi: 10.3390/v18010137 (PMC12846518; doi:10.3390/v18010137)

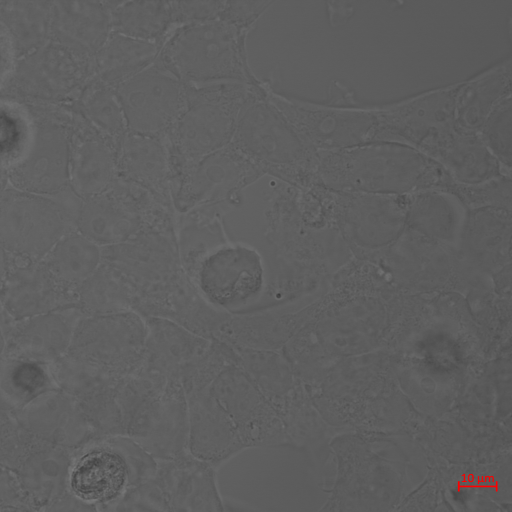

Supplement: Supplementary file 1 [file viruses-18-00137-s001.zip › Supplementary file 2 (original images)/Immunofluorescence confocal microscopy/S-dCT19 (a-gl UTRs)_after permeabilization/Bright field_3117_perm_2_c2.tif]

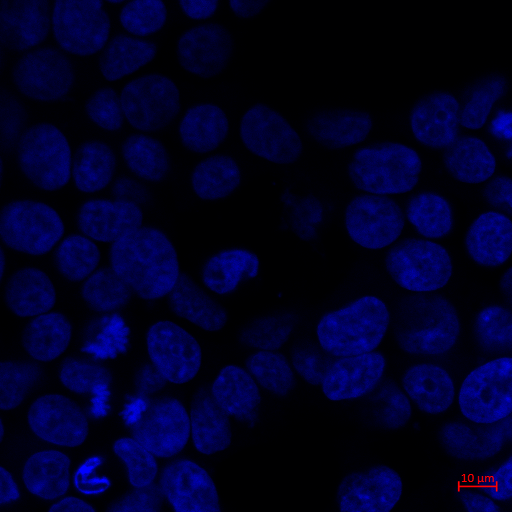

Supplement: Supplementary file 1 [file viruses-18-00137-s001.zip › Supplementary file 2 (original images)/Immunofluorescence confocal microscopy/S-dCT19 (a-gl UTRs)_after permeabilization/Hoechst 33342_3117_perm_2_c4.tif]

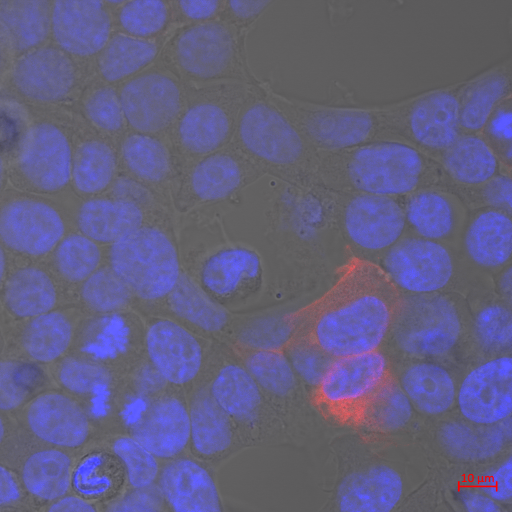

Supplement: Supplementary file 1 [file viruses-18-00137-s001.zip › Supplementary file 2 (original images)/Immunofluorescence confocal microscopy/S-dCT19 (a-gl UTRs)_after permeabilization/Merge_3117_perm_2_c1+2+4.tif]

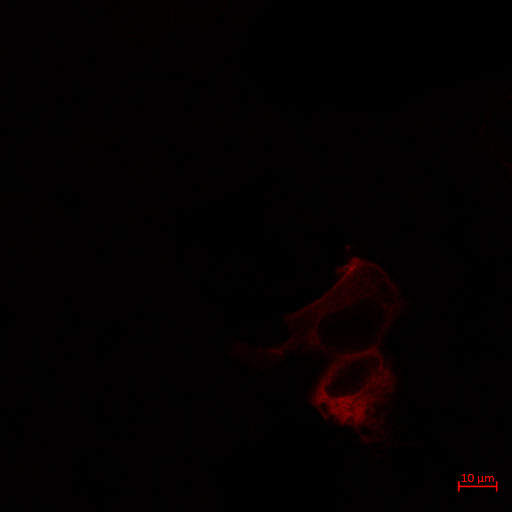

Supplement: Supplementary file 1 [file viruses-18-00137-s001.zip › Supplementary file 2 (original images)/Immunofluorescence confocal microscopy/S-dCT19 (a-gl UTRs)_after permeabilization/S-protein_3117_perm_2_c1.tif]

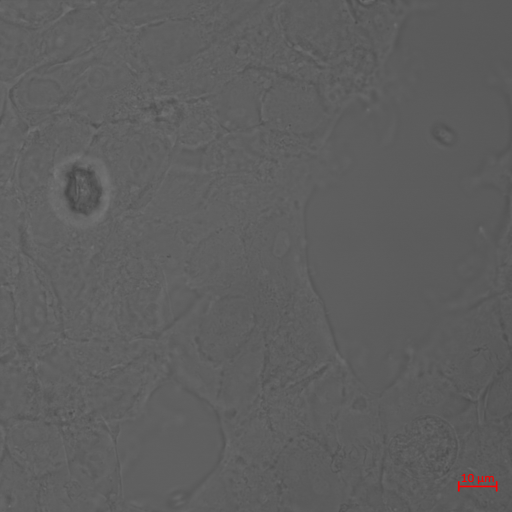

Supplement: Supplementary file 1 [file viruses-18-00137-s001.zip › Supplementary file 2 (original images)/Immunofluorescence confocal microscopy/S-dCT19 (a-gl UTRs)_surface/Bright field_3117_surface_2_c2.tif]

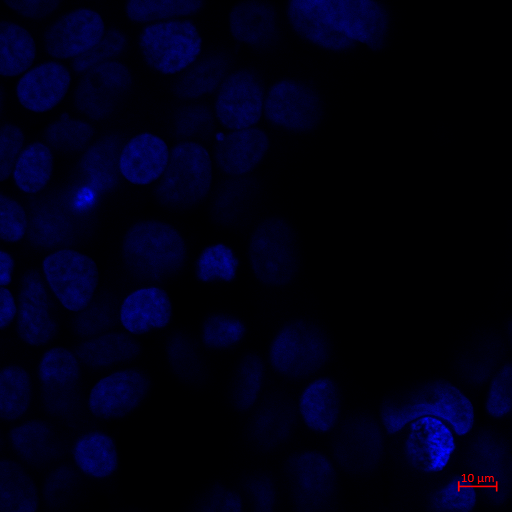

Supplement: Supplementary file 1 [file viruses-18-00137-s001.zip › Supplementary file 2 (original images)/Immunofluorescence confocal microscopy/S-dCT19 (a-gl UTRs)_surface/Hoechst 33342_3117_surface_2_c4.tif]

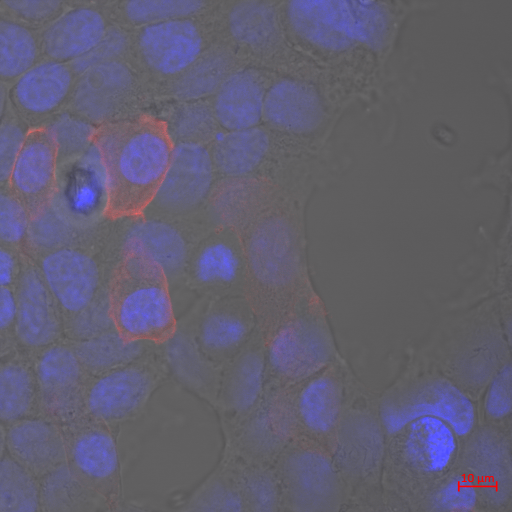

Supplement: Supplementary file 1 [file viruses-18-00137-s001.zip › Supplementary file 2 (original images)/Immunofluorescence confocal microscopy/S-dCT19 (a-gl UTRs)_surface/Merge_3117_surface_2_c1+2+4.tif]

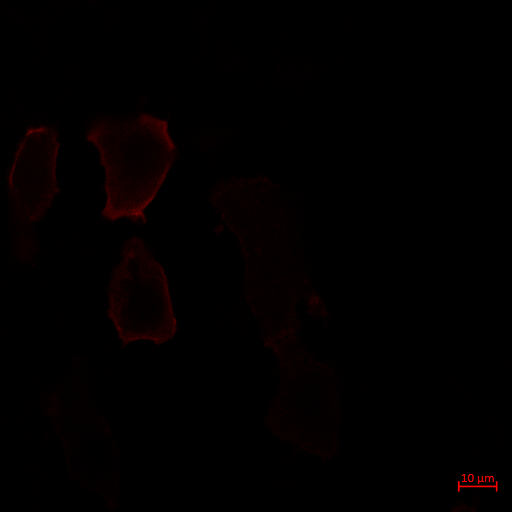

Supplement: Supplementary file 1 [file viruses-18-00137-s001.zip › Supplementary file 2 (original images)/Immunofluorescence confocal microscopy/S-dCT19 (a-gl UTRs)_surface/S-protein_3117_surface_2_c1.tif]

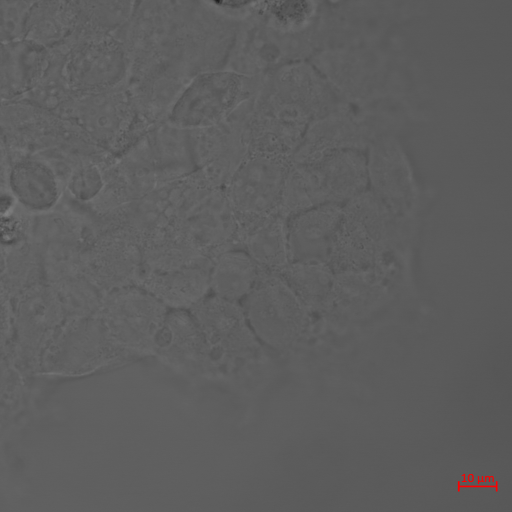

Supplement: Supplementary file 1 [file viruses-18-00137-s001.zip › Supplementary file 2 (original images)/Immunofluorescence confocal microscopy/S-dCT19CD47-СT-tail (a-gl UTRs HuR-BS)_after permeabilization/Bright field_4002_perm_1_c2.tif]

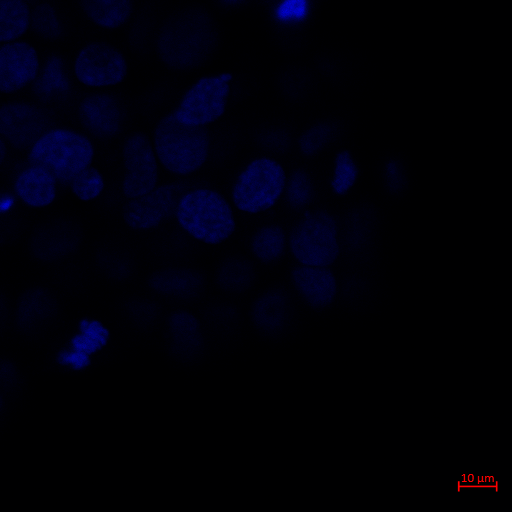

Supplement: Supplementary file 1 [file viruses-18-00137-s001.zip › Supplementary file 2 (original images)/Immunofluorescence confocal microscopy/S-dCT19CD47-СT-tail (a-gl UTRs HuR-BS)_after permeabilization/Hoechst 33342_4002_perm_1_c4.tif]

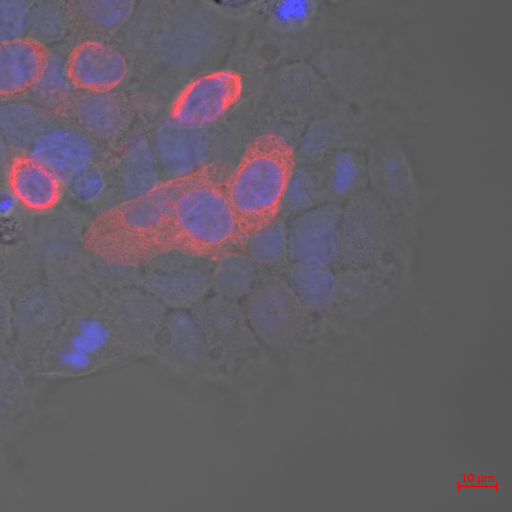

Supplement: Supplementary file 1 [file viruses-18-00137-s001.zip › Supplementary file 2 (original images)/Immunofluorescence confocal microscopy/S-dCT19CD47-СT-tail (a-gl UTRs HuR-BS)_after permeabilization/Merge_4002_perm_1_c1+2+4.tif]

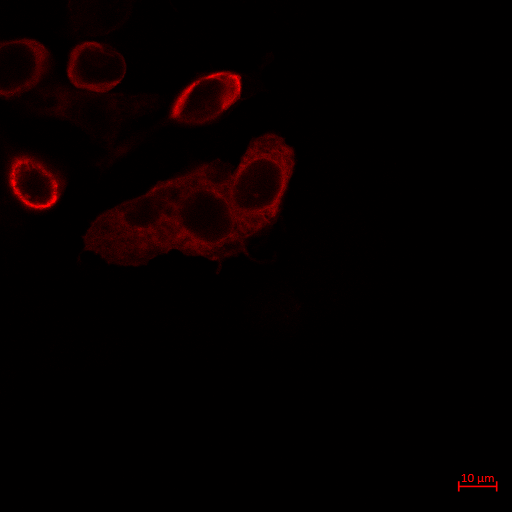

Supplement: Supplementary file 1 [file viruses-18-00137-s001.zip › Supplementary file 2 (original images)/Immunofluorescence confocal microscopy/S-dCT19CD47-СT-tail (a-gl UTRs HuR-BS)_after permeabilization/S-protein_4002_perm_1_c1.tif]

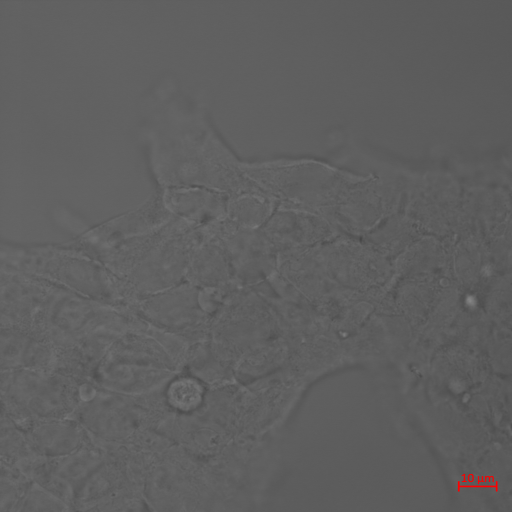

Supplement: Supplementary file 1 [file viruses-18-00137-s001.zip › Supplementary file 2 (original images)/Immunofluorescence confocal microscopy/S-dCT19CD47-СT-tail (a-gl UTRs HuR-BS)_surface/Bright field_4002_surface_1_T PMT-T1.tif]

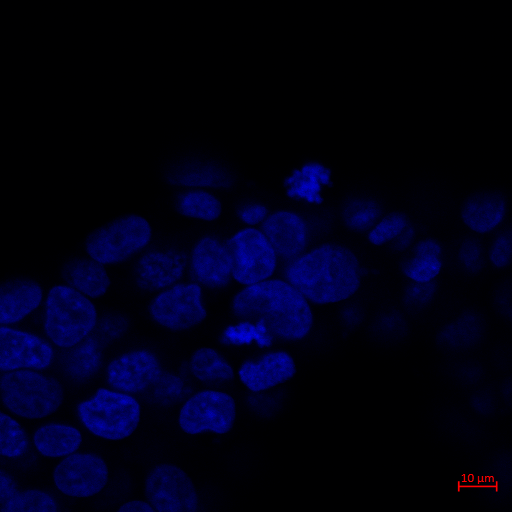

Supplement: Supplementary file 1 [file viruses-18-00137-s001.zip › Supplementary file 2 (original images)/Immunofluorescence confocal microscopy/S-dCT19CD47-СT-tail (a-gl UTRs HuR-BS)_surface/Hoechst 33342_4002_surface_1_Ch1-T3.tif]

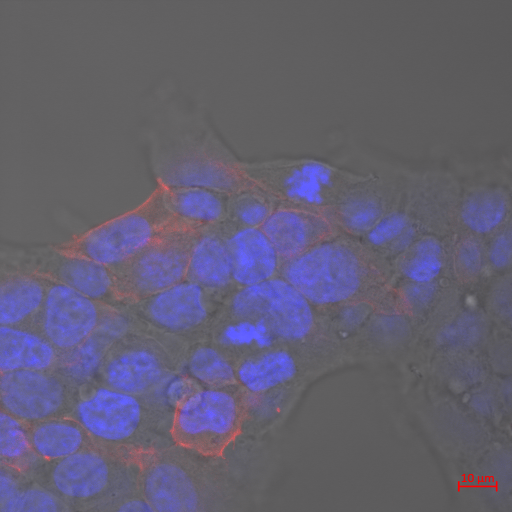

Supplement: Supplementary file 1 [file viruses-18-00137-s001.zip › Supplementary file 2 (original images)/Immunofluorescence confocal microscopy/S-dCT19CD47-СT-tail (a-gl UTRs HuR-BS)_surface/Merge_4002_surface_1.tif]

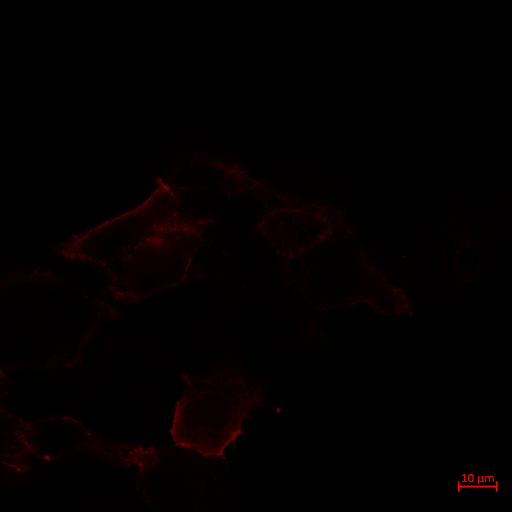

Supplement: Supplementary file 1 [file viruses-18-00137-s001.zip › Supplementary file 2 (original images)/Immunofluorescence confocal microscopy/S-dCT19CD47-СT-tail (a-gl UTRs HuR-BS)_surface/S-protein_4002_surface_1_Ch3-T1.tif]

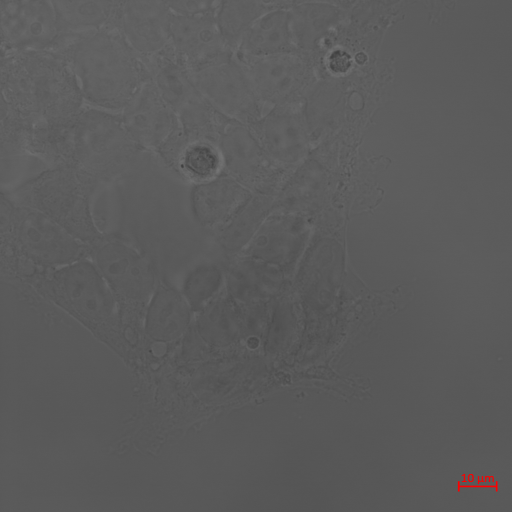

Supplement: Supplementary file 1 [file viruses-18-00137-s001.zip › Supplementary file 2 (original images)/Immunofluorescence confocal microscopy/S-FL (a-gl UTRs)_after permeabilization/Bright field_3231_perm_2_c2.tif]

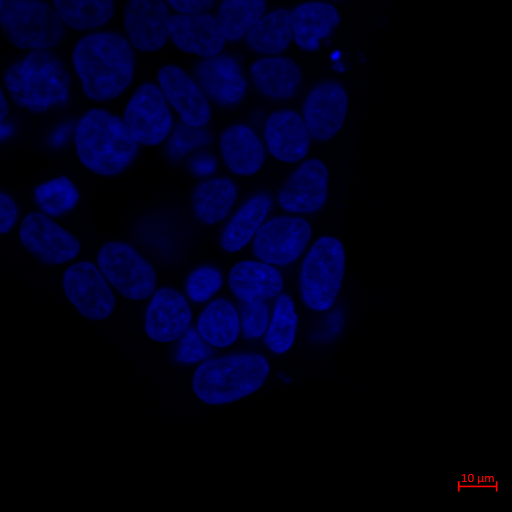

Supplement: Supplementary file 1 [file viruses-18-00137-s001.zip › Supplementary file 2 (original images)/Immunofluorescence confocal microscopy/S-FL (a-gl UTRs)_after permeabilization/Hoechst 33342_3231_perm_2_c4.tif]

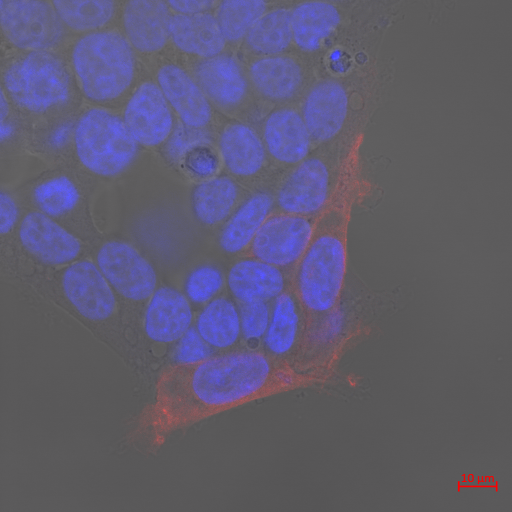

Supplement: Supplementary file 1 [file viruses-18-00137-s001.zip › Supplementary file 2 (original images)/Immunofluorescence confocal microscopy/S-FL (a-gl UTRs)_after permeabilization/Merge_3231_perm_2_c1+2+4.tif]

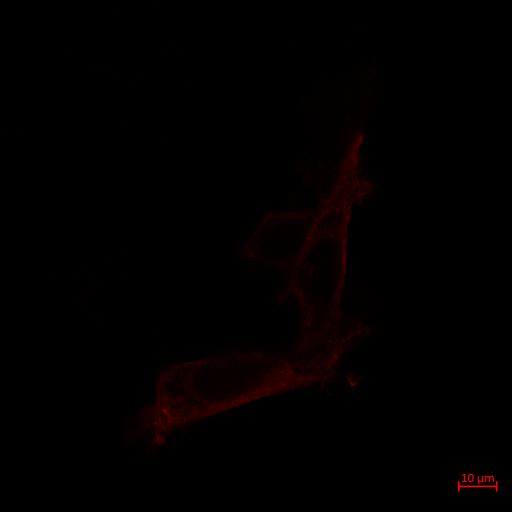

Supplement: Supplementary file 1 [file viruses-18-00137-s001.zip › Supplementary file 2 (original images)/Immunofluorescence confocal microscopy/S-FL (a-gl UTRs)_after permeabilization/S-protein_3231_perm_2_c1.tif]

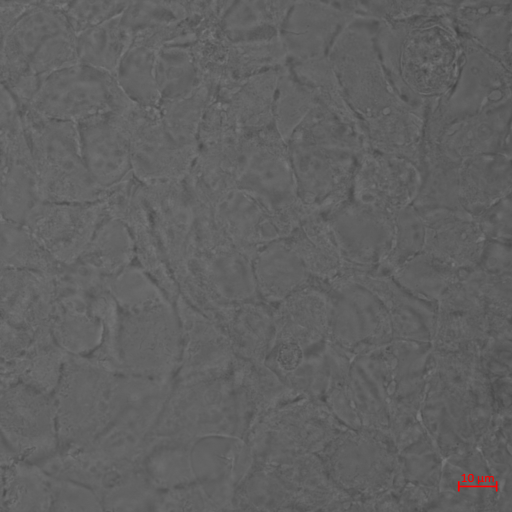

Supplement: Supplementary file 1 [file viruses-18-00137-s001.zip › Supplementary file 2 (original images)/Immunofluorescence confocal microscopy/S-FL (a-gl UTRs)_surface/Bright field_3231_surface_1_T PMT-T1.tif]

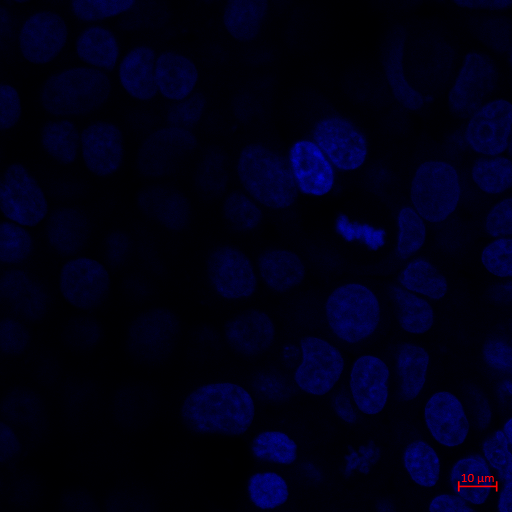

Supplement: Supplementary file 1 [file viruses-18-00137-s001.zip › Supplementary file 2 (original images)/Immunofluorescence confocal microscopy/S-FL (a-gl UTRs)_surface/Hoechst 33342_3231_surface_1_Ch1-T3.tif]

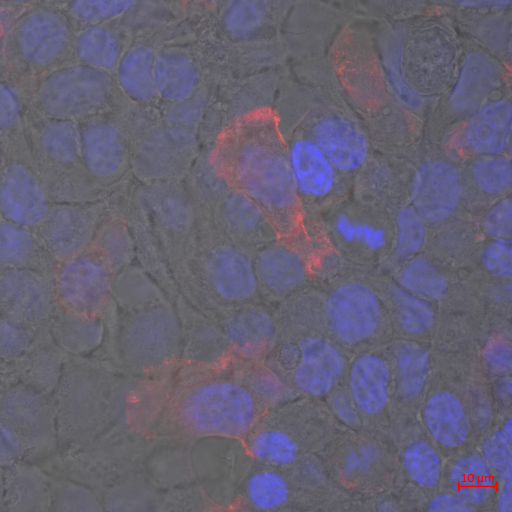

Supplement: Supplementary file 1 [file viruses-18-00137-s001.zip › Supplementary file 2 (original images)/Immunofluorescence confocal microscopy/S-FL (a-gl UTRs)_surface/Merge_3231_surface_1.tif]

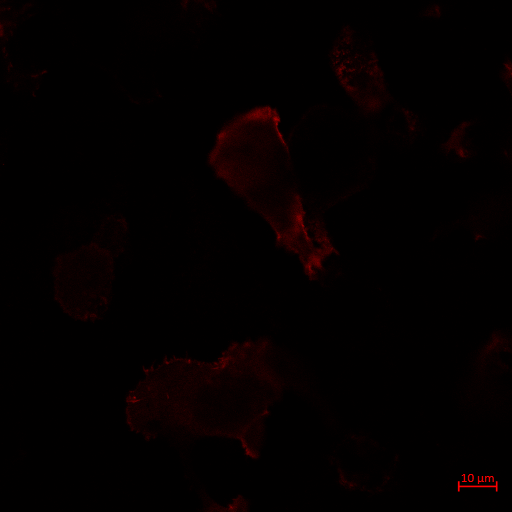

Supplement: Supplementary file 1 [file viruses-18-00137-s001.zip › Supplementary file 2 (original images)/Immunofluorescence confocal microscopy/S-FL (a-gl UTRs)_surface/S-protein_3231_surface_1_Ch3-T1.tif]
